# Half-Sandwich Rhodium Complexes with Releasable N-Donor Monodentate Ligands: Solution Chemical Properties and the Possibility for Acidosis Activation

**DOI:** 10.3390/pharmaceutics15020356

**Published:** 2023-01-20

**Authors:** János P. Mészáros, Wolfgang Kandioller, Gabriella Spengler, Alexander Prado-Roller, Bernhard K. Keppler, Éva A. Enyedy

**Affiliations:** 1 MTA-SZTE Lendület Functional Metal Complexes Research Group, University of Szeged, Dóm tér 7, H-6720 Szeged, Hungary; 2 Department of Inorganic and Analytical Chemistry, Interdisciplinary Excellence Centre, University of Szeged, Dóm tér 7, H-6720 Szeged, Hungary; 3 Institute of Inorganic Chemistry, Faculty of Chemistry, University of Vienna, Währinger Str. 42, A-1090 Vienna, Austria; 4 Research Cluster “Translational Cancer Therapy Research”, University of Vienna, Währinger Str. 42, A-1090 Vienna, Austria; 5 Department of Medical Microbiology, Albert Szent-Györgyi Health Center and Albert Szent-Györgyi Medical School, University of Szeged, Semmelweis utca 6, H-6725 Szeged, Hungary

**Keywords:** piano-stool, pentamethylcyclopentadienyl, anticancer, formation constant, organometallic

## Abstract

Cancer chemotherapeutics usually have serious side effects. Targeting the special properties of cancer and activation of the anticancer drug in the tumor microenvironment in situ may decrease the intensity of the side effects and improve the efficacy of therapy. In this study, half-sandwich Rh complexes are introduced, which may be activated at the acidic, extracellular pH of the tumor tissue. The synthesis and aqueous stability of mixed-ligand complexes with a general formula of [Rh(η^5^-Cp*)(N,N/O)(N)]^2+/+^ are reported, where (N,N/O) indicates bidentate 8-quinolate, ethylenediamine and 1,10-phenanthroline and (N) represents the releasable monodentate ligand with a nitrogen donor atom. UV-visible spectrophotometry, ^1^H NMR, and pH-potentiometry were used to determine the protonation constants of the monodentate ligands, the proton dissociation constants of the coordinated water molecules in the aqua complexes, and the formation constants of the mixed-ligand complexes. The obtained data were compared to those of the analogous Ru(η^6^-*p*-cymene) complexes. The developed mixed-ligand complexes were tested in drug-sensitive and resistant colon cancer cell lines (Colo205 and Colo320, respectively) and in four bacterial strains (Gram-positive and Gram-negative, drug-sensitive, and resistant) at different pH values (5–8). The mixed-ligand complexes with 1-methylimidazole displayed sufficient stability at pH 7.4, and their activation was found in cancer cells with decreasing pH; moreover, the mixed-ligand complexes demonstrated antimicrobial activity in Gram-positive and Gram-negative bacteria, including the resistant MRSA strain. This study proved the viability of incorporating releasable monodentate ligands into mixed-ligand half-sandwich complexes, which is supported by the biological assays.

## 1. Introduction

Selectivity is a crucial factor in the treatment of cancer since the active component should distinguish the tumor tissue from the healthy one. Insufficient selectivity can be responsible for serious side effects, which usually occur during chemotherapy and have a great impact on patient compliance [1,2]. In order to improve selectivity, the specific characteristics of the cancer cells and their microenvironment should be targeted. Metabolic reprogramming in cancer cells induces the intensification of glycolysis [3,4]. This is the so-called Warburg effect, which is connected to the higher proliferation rate and the hypoxic conditions developed far from the vessels [4,5]. As a result, the amount of lactate and H^+^ ions increases; however, they are pumped out of the cell due to the upregulation of acid-extruding transmembrane ion transporters [5]. While the intracellular pH increases even above that of healthy cells, the extracellular pH decreases (between 6–6.7). Several studies have addressed an excellent idea of using proton pump inhibitors for targeting tumor pH homeostasis [6]. These compounds exhibited antimetastatic and/or cytotoxic effects in various cancer cells in vitro and in vivo, and they enhanced the efficacy of coadministered chemotherapeutics. Acidosis of the tumor tissue is the special feature that is also targeted by the title compounds, and the possible activation at acidic conditions was tested.

**Chart 1:**
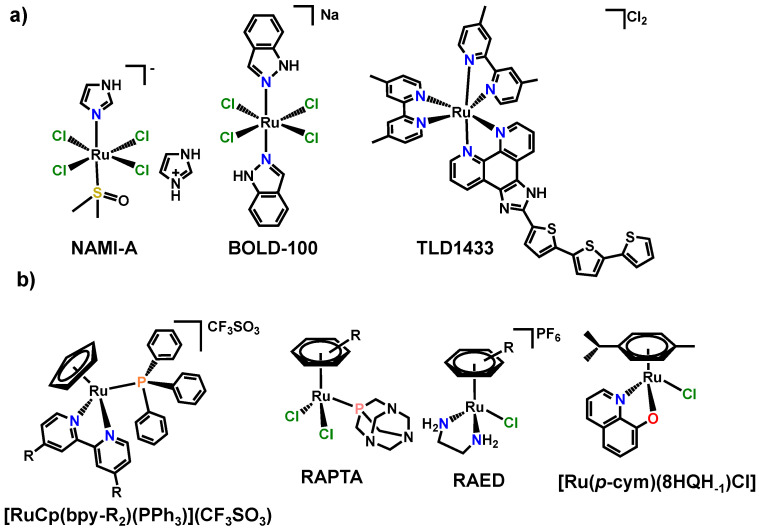
Structures of (**a**) octahedral and (**b**) half-sandwich Ru complexes with promising anticancer activity.

Cisplatin is the best example of the use of inorganic compounds and complexes of transition metals in cancer chemotherapy. The discovery of its inhibitory effect on cell division and its clinical approval were important steps in the history of medicinal inorganic chemistry [7]. It is a robust chemotherapeutic drug that is used in the treatment of a wide spectrum of cancers; it is particularly effective against testicular tumors, where the cure rates are above 95% [7]. Although platinum complexes (cisplatin, carboplatin, and oxaliplatin) are used in clinics worldwide, their use is accompanied by severe side effects and intrinsic/acquired platinum resistance, which impede therapeutic success [7]. There is a need for more effective and selective compounds. For that purpose, ruthenium is the most extensively studied platinum group metal ion, and three complexes with a Ru(II/III) center (namely: NAMI-A, BOLD-100, and TLD1433, Chart 1a) have entered into clinical trials so far [8,9]. Among these, BOLD-100 was granted orphan drug designation in the treatment of gastric cancer by the FDA [10]. In these complexes, the ligands have nitrogen donor atom(s). In contrast, in TLD1433, the metal center is surrounded by bidentate (N,N) donor ligands, and in NAMI-A and BOLD-100, monodentate ligands are present (imidazole and indazole, respectively).

The organometallic compounds of ruthenium are also of interest since they showed promising anticancer effects in the preclinical stage; some drug candidates are highlighted in Chart 1b. Half-sandwich Ru(II) complexes containing cyclopentadienyl and triphenylphosphine (PPh_3_) ligands showed cytotoxic effects in vivo [11], while another group, abbreviated as RAPTA (ruthenium arene 1,3,5-triaza-7-phosphaadamantane) complexes, has antimetastatic activity in vivo [12]. In these complexes, 1,3,5-triaza-7-phosphaadamantane (PTA) or its derivatives are used as monodentate ligands, having a phosphorous donor atom. The most studied half-sandwich complexes have the general structure [M(arene)(X,Y)(Z)], in which M is a platinum group metal ion: (Ru(II), Os(II), Rh(III), and Ir(III)); arene is a neutral benzene derivative (mostly for Ru(II) and Os(II)) or pentamethylcyclopentadienyl anion (Cp*) derivative (mostly for Rh(III) and Ir(III)); (X,Y) is a bidentate ligand, and Z is a monodentate ligand, usually having a role in the leaving group (halide ion or H_2_O) [13,14,15,16]. Among these compounds, RAED (ruthenium arene ethylenediamine) complexes, bearing ethylenediamine (en) as bidentate (N,N) donor ligand, showed excellent cytotoxicity in vitro (IC_50_ ≤ 10 μM) [17]. Despite the strong anticancer effect of the RAED complexes, their Rh(III)-containing analogs proved to be inactive [18]. Surprisingly, the Rh(III) complex turned out to be effective in a wide range of cancer cell lines, when 1,10-phenanthroline (phen) is in the role of the bidentate ligand [18,19], while the Ru(II) analog showed decreased cytotoxicity [19,20]. The (N,O) donor atoms bearing 8-hydroxyquinoline (8HQ) co-ordinate with both [Ru(η^6^-*p*-cymene)(H_2_O)_3_]^2+^ ([Ru(*p*-cym)(H_2_O)_3_]^2+^) and [Rh(η^5^-Cp*)(H_2_O)_3_]^2+^ ([RhCp*(H_2_O)_3_]^2+^) in its deprotonated form (8HQH_−1_); its complexes have promising cytotoxic activity (IC_50_ ≤ 10 μM) in different cancer cell lines [21]. The stability of [M(arene)(X,Y)(Z)] complexes in an aqueous solution is highly dependent on the bidentate ligand. Ligands with (O,O) donors are set from less stable complexes than those with (N,O) or (N,N) donor atoms [22]. The reactivity and the mechanism of action of the complex can be controlled by the use of the proper monodentate ligand. The most commonly used chloride ion is present in the biofluids; thus, the chloride ion concentration affects the reactivity of the complex. The actual chloride ion concentration determines the fractions of the chlorinated (Z = Cl^−^, nonactivated form) and the aqua complexes (Z = H_2_O, activated form) [23]. The complex remains intact for a longer time when the iodide ion [23] or a monodentate ligand with a phosphorous or sulfur donor atom is in that position [12,18].

**Chart 2:**
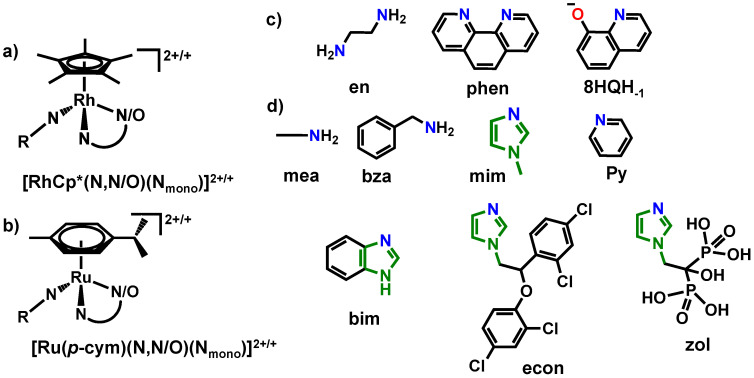
General structure of the mixed-ligand (**a**) RhCp* and (**b**) Ru (*p*-cym) complexes. (**c**) The used bidentate ligands with (N,N) and (N,O) donor atoms and (**d**) monodentate N-donor ligands. (The counter ions are Cl^−^, CF_3_SO_3_^−^, or BPh_4_^−^, as indicated in the experimental section for each complex).

There are examples reported for the design of the leaving groups that are activated by acidosis. Tethered Ru(II) and Ir(III) complexes were developed, in which the monodentate leaving group was covalently bound to the arene ring. The donor atom was part of a functional group capable of protonation (carboxylate, amino group, or pyridine ring nitrogen), and the co-ordination processes to the metal center were followed as a function of pH [24,25,26,27]. The tethered complexes showed pH-responsive ring opening and closure; however, in vitro tests showed decreased cytotoxic activity compared to the nontethered analogs. Although activation may happen in the decreased extracellular pH, the higher intracellular pH may cause deactivation by the induction of ring closure. As a demonstration of the viability of the tethered complex design, the combination of (C,N) donor bidentate ligands and a tethered pyridine ring on a Cp * moiety resulted in highly active and selective Ir(III) complexes [25]. Herein, we present RhCp* complexes bearing (N,N) or (N,O) bidentate ligands and nontethered releasable (N_mono_) monodentate leaving groups that can be activated by acidosis (Chart 2).

## 2. Materials and Methods

### 2.1. Chemicals

All solvents were of analytical grade and were used without further purification. Ethylenediamine, 1,10-phenanthroline, 8-hydroxyquinoline, pyridine (Py), 1-methylimidazole (mim), benzimidazole (bim), methylamine (mea) hydrochloride, benzylamine (bza), zoledronic acid (zol) monohydrate, econazole (econ) nitrate, [RhCp*(*μ*-Cl)Cl]_2_, [Ru(*p*-cym)(*μ*-Cl)Cl]_2_, doxorubicin, KCl, HCl, KOH, 4,4-dimethyl-4-silapentane-1-sulfonic acid (DSS), NaH_2_PO_4_, Na_2_HPO_4_, KH_2_PO_4_, Na(CF_3_SO_3_), sodium tetraphenylborate, 3-(4,5-dimethylthiazol-2-yl)-2,5-diphenyltetrazolium bromide (MTT), sodium dodecyl sulfate (SDS), Roswell Park Memorial Institute (RPMI) 1640 medium, fetal bovine serum, Mueller Hinton Broth, *L*-glutamine, sodium pyruvate, 4-(2-hydroxyethyl)-1-piperazineethanesulfonic acid (HEPES), Trypsin-Versene reagent, 2,5-bis-(5-tert-butyl-2-benzoxazol-2-yl)-thiophenone (BBOT), and sulfanilamide were purchased from Sigma-Aldrich (Merck KGaA, Darmstadt, Germany) in puriss quality. Ultrapure Milli-Q water was used for sample preparation. Mixed-ligand complexes (complexes 1–6) were synthesized for structural comparison (see synthesis in Section 2.2.1).

The exact concentration of ligand stock solutions and the protonation constants were determined by pH-potentiometric titrations. The computer program Hyperquad2013 was used for calculations [28]. The aqueous [RhCp*(H_2_O)_3_]^2+^ and [Ru(*p*-cym)(H_2_O)_3_]^2+^ stock solutions were obtained by dissolving the dimeric precursor in water, and their exact concentrations were determined by pH-potentiometric titrations. Stock solution of benzimidazole was prepared on a weight-in-volume basis in water, and the concentration was checked by quantitative ^1^H NMR using known amounts of Py as the internal standard since its limited water solubility hindered the use of pH-potentiometry.

A modified phosphate-buffered saline (PBS’) was used as a medium for the buffered samples. PBS’ contains 12 mM Na_2_HPO_4_, 3 mM KH_2_PO_4_, 1.5 mM KCl, and 100.5 mM NaCl. The concentrations of the K^+^, Na^+^, and Cl^−^ ions were equal to those found in human blood serum. The pH was set at pH = 6.0 or 7.4 by drops of H_3_PO_4_ and KOH solutions. Phosphate does not interact with these organometallic cations and most of their complexes, based on our preliminary studies, except with [Ru(arene)(en)(H_2_O)]^2+^ [19]. This interaction is fairly weak at pH 7.4 and does not disturb the co-ordination of the monodentate nitrogen donor ligands.

Samples for biological studies were mixed prior to the measurement. The mixed-ligand complexes were formed in situ when the monodentate ligand was added to the aqua complex. The use of these samples was necessary since the synthesized mixed-ligand complexes (synthesis described in Section 2.2.1) have different counter ions, and this difference may have a tremendous effect on the anticancer effect, as was the case when introduced in some half-sandwich Ir(III) complexes [29]. Although the use of tetraphenylborate anion was successful in the induction of single-crystal formation, it has a disadvantageous effect on anticancer activity and on solubility (in MeOH and in water as well). Since this work focuses on the effect of mixed-ligand complexes and finding the optimal bidentate ligand–monodentate ligand combination for RhCp* complexes, a simple counterion, chloride ion was our choice for these studies. To prove that these samples contain the mixed-ligand complexes under the given conditions (5 mM concentration, 50% (*v*/*v*) ethanol/phosphate buffer (pH = 7.4)), the addition of the econazole monodentate ligand to the [RhCp*(8HQH_−1_)(H_2_O)]^+^ complex (Figure S1) in different equivalents was followed by UV-vis spectroscopy. The spectra at equimolar concentrations and at higher econazole-to-[RhCp*(8HQH_−1_)(H_2_O)]^+^ ratios were identical. This means that the equimolar solution (which was used for anticancer and antibacterial studies) contained the already desired [RhCp*(8HQH_−1_)(econ)]^+^ complex exclusively.

### 2.2. Synthesis and Characterization of Complexes

#### 2.2.1. General Synthesis of Mixed-Ligand Complexes

**Scheme 1:**
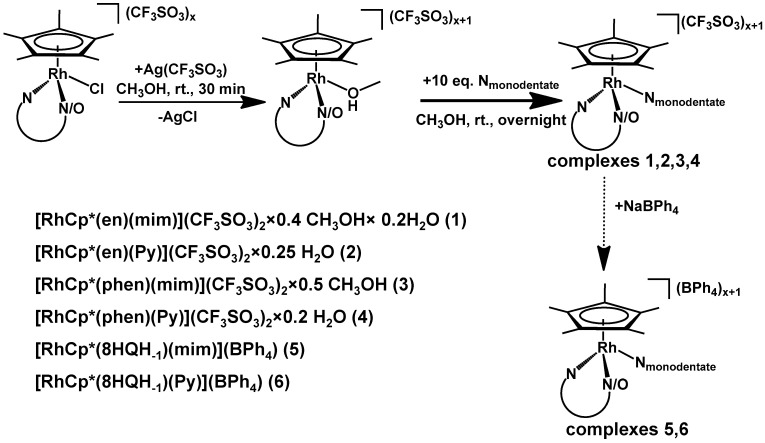
Synthetic steps for the preparation of the mixed-ligand half-sandwich RhCp* complexes.

The synthetic steps are shown in Scheme 1. The first step was the synthesis of the [RhCp*(en)Cl](CF_3_SO_3_), [RhCp*(phen)Cl](CF_3_SO_3_), and [RhCp*(8HQH_−1_)Cl] complexes, which was performed following previously published methods [21,30]. Then, 0.10–0.15 mmol of the complex was dissolved in ~3 mL methanol, and chloride ion abstraction was performed by the reaction with an equivalent amount of Ag(CF_3_SO_3_) (light exclusion, 30 min, room temperature) followed by the filtration of AgCl. The addition of 10 equivalents of the monodentate ligand (pyridine or 1-methylimidazole) immediately caused a slight color change from orange to pale yellow. Afterward, the reaction mixture was stirred overnight at room temperature and was concentrated in vacuo, and the formed product was separated by filtration. The mixed-ligand complexes were purified by recrystallization from methanol and washed with ether or *n*-hexane. The optimal crystallization and purification of the [RhCp*(8HQH_-1_)(Py/mim)]^+^ complexes could be reached only by the use of tetraphenylborate anions. Single crystals suitable for structure determination were grown from the solution of the synthesized complexes (1–6). The solids were dissolved in MeOH, and, from the saturated solutions, single crystals were grown with two methods: a vapor diffusion method (methanol (MeOH)–diethylether) and slow evaporation of the solvent (MeOH–CH_2_Cl_2_ (1:1) mixture) for [RhCp*(phen)(mim)](BPh_4_)_2_. In parallel with the original CF_3_SO_3_^−^-containing samples, portions of sodium tetraphenylborate were also used to induce crystallization, which was successful for the three mixed-ligand complexes.

The Supplementary Material contains the ^1^H and ^13^C NMR spectra of **complexes 1–6** (Figures S2–S14). It is worthwhile to mention that the co-ordination of aromatic nitrogen atoms (Py, phen) promotes the deuteration of the Cp* ligand. Signs of deuterated methyl groups were visible in both the ^1^H and ^13^C NMR spectra, highlighted in Figures S4, S5 and S8–S10. Similar behavior was found in our previous work [19], and the mechanism was investigated by mass spectrometry and DFT calculations as well [31].

**Complex 1**: [RhCp*(en)Cl](CF_3_SO_3_) (48 mg, 0.1 mmol), Ag(CF_3_SO_3_) (25.7 mg, 0.1 mmol) and 1-methylimidazole (80 μL, 1 mmol); yield: 54.4 mg (78%). Elemental analysis calculated for C_18_H_29_F_6_N_4_O_6_RhS_2_ × 0.4 MeOH × 0.2 H_2_O: C 31.65%, H 4.35%, N 8.20% O 14.64%, S 9.39. Found: C 31.71%, H 4.14%, N 7.82%, O 14.44%, S 8.77%.

^1^H NMR (CD_3_OD, ppm, 500 MHz, standard: δ 3.31 (solvent residual peak)): δ 8.08 (s, 1H, mim-**H^2^**), δ 7.40 (s, 1H, mim-**H^4^**), δ 7.19 (s, 1H, mim-**H^5^**), δ 3.87 (s, 3H, mim-**H^methyl^**), δ 2.58–2.64 (m, 2H, en-**H^methylene^**), δ 2.25–2.30 (m, 2H, en-**H^methylene^**), δ 1.70 (s, 15H, Cp*-**H^methyl^**).

^13^C NMR (CD_3_OD, ppm, 125 MHz, standard: δ 49.00 (solvent residual peak)): δ 141.83 (s, mim-**C^2^**), δ 129.72 (s, mim-**C^4^**), δ 125.17 (s, mim-**C^5^**), δ 121.79 (q, J_13C–19F_ = 318.5 Hz, triflate-**CF_3_**), δ 97.49 (d, J_13C–103Rh_ = 7.7 Hz, Cp*-**C^ring^**), δ 35.05 (s, mim-**C^methyl^**), δ 8.78 (s, Cp*-**C^methyl^**). Coupling with ^103^Rh is highlighted in Figure S3.

**Complex 2**: [RhCp*(en)Cl](CF_3_SO_3_) (72 mg, 0.15 mmol), Ag(CF_3_SO_3_) (39 mg, 0.15 mmol) and pyridine (121.3 μL, 1.5 mmol); yield: 86 mg (85%). Elemental analysis calculated for C_19_H_28_F_6_N_3_O_6_RhS_2_ × 0.25 H_2_O: C 33.56%, H 4.22%, N 6.18%, O 14.71%, S 9.43%. Found: C 33.14%, H 4.26%, N 5.95%, O 14.52%, S 9.27%.

^1^H NMR (CD_3_OD, ppm, 500 MHz, standard: δ 3.31 (solvent residual peak)): δ 8.69 (d, J = 5.0 Hz, 2H, Py-**H^2,6^**), δ 8.19 (tt, J = 7.7 Hz, 1.49 Hz, 1H, Py-**H^4^**), δ 7.76 (t, J = 7.0 Hz, 2H, Py-**H^3,5^**), δ 2.67–2.71 (m, 2H, en-**H^methylene^**), δ 2.27–2.32 (m, 2H, en-**H^methylene^**), δ 1.68 (s, 15H, Cp*-**H^methyl^**). Deuteration of Cp* is visible and highlighted in the spectrum in Figure S4.

^13^C NMR (CD_3_OD, ppm, 125 MHz, standard: δ 49.00 (solvent residual peak)): δ 154.15 (s, Py-**C^2,6^**), δ 141.56 (s, Py-**C^4^**), δ 129.12 (s, Py-**C^3,5^**), δ 121.79 (q, J_13C–19F_ = 318.5 Hz, triflate-**CF_3_**), δ 98.16 (d, J_13C–103Rh_ = 6.9 Hz, Cp*-**C^ring^**), δ 8.71 (s, Cp*-**C^methyl^**). Deuteration of Cp* and coupling with ^19^F (triflate) are visible and highlighted in the spectrum in Figure S5.

**Complex 3:** The structure was determined from the tetraphenylborate salt, which formed bigger crystals than with triflate counter ion. Elemental analysis and NMR spectra were obtained for the triflate salt. [RhCp*(phen)Cl](CF_3_SO_3_) (90.4 mg, 0.15 mmol), Ag(CF_3_SO_3_) (38.5 mg, 0.15 mmol) and 1-methylimidazole (119.6 μL, 1.5 mmol); yield: 82.3 mg (67%). The structure was determined for the tetraphenylborate salt, which formed bigger crystals than with triflate counter ion. Elemental analysis calculated for C_28_H_29_F_6_N_4_O_6_RhS_2_×0.5 MeOH: C 42.02%, H 3.84%, N 6.88%, O 12.63%, S 7.87. Found: C 41.91%, H 3.59%, N 6.68%, O 12.36%, S 7.45.

^1^H NMR (CD_3_OD, ppm, 500 MHz, standard: δ 3.31 (solvent residual peak)): δ 9.68 (dd, J = 5.2 Hz, 1.18 Hz, 2H, phen-**H^2,9^**), δ 8.96 (dd, J = 8.2 Hz, 1.3 Hz, 2H, phen-**H^4,7^**), δ 8.34 (dd, J = 8.2 Hz, 5.2 Hz, 2H, phen-**H^3,8^**), δ 8.25 (s, 2H, phen-**H^5,6^**), δ 7.74 (s, 1H, mim-**H^2^**), δ 7.06 (t, J = 1.5 Hz, 1H, mim-**H^4^**), δ 6.85 (t, J = 1.5 Hz, 1H, mim-**H^5^**), δ 3.56 (s, 3H, mim-**H^methyl^**), δ 1.77 (s, 15H, Cp*-**H^methyl^**). Deuteration of Cp* is visible in the spectrum in Figure S6.

^13^C NMR (CD_3_OD, ppm, 125 MHz, standard: δ 49.00 (solvent residual peak)): δ 153.88 (s, phen-**C^2,9^**), δ 146.76 (s, phen-**C^4a,6a^**), δ 141.46 (s, phen-**C^4,7^**), δ 140.39 (s, mim-**C^2^**), δ 132.54 (s, phen-**C^10a,10b^**), δ 129.33 (s, phen-**C^3,8^**), δ 128.93 (s, phen-**C^5,6^**), δ 128.81 (s, mim-**C^4^**), δ 125.04 (s, mim-**C^5^**), δ 121.79 (q, J_13C–19F_ = 318.5 Hz, triflate-**CF_3_**), δ 100.33 (d, J_13C–103Rh_ = 7.7 Hz, Cp*-**C^ring^**), δ 34.87 (s, mim-**C^methyl^**), δ 8.76 (s, Cp*-**C^methyl^**). Deuteration of Cp* is visible and highlighted in Figures S7 and S8.

**Complex 4**: [RhCp*(phen)Cl]Cl (90.4 mg, 0.15 mmol), Ag(CF_3_SO_3_) (38.5 mg, 0.15 mmol) and pyridine (121.3 μL, 1.5 mmol); yield: 80.8 mg (66%). Elemental analysis calculated for C_29_H_28_F_6_N_3_O_6_RhS_2_×0.2H_2_O: C 43.58%, H 3.58%, N 5.26%, O 12.41%, S 8.02%. Found: C 43.49%, H 3.53%, N 5.10%, O 12.37%, S 7.43%.

^1^H NMR (CD_3_OD, ppm, 500 MHz, standard: δ 3.31 (solvent residual peak)): δ 9.76 (dd, J = 5.2 Hz, 0.87 Hz, 2H, phen-**H^2,9^**), δ 8.99 (dd, J = 8.3 Hz, 1.2 Hz, 2H, phen-**H^4,7^**), δ 8.49 (d, J = 5.2 Hz, 2H, Py-**H^2,6^**), δ 8.40 (dd, J = 8.2 Hz, 5.2 Hz, 2H, phen-**H^3,8^**), δ 8.25 (s, 2H, phen-**H^5,6^**), δ 7.91 (tt, J = 7.7 Hz, 1.4 Hz, 1H, Py-**H^4^**), δ 7.46 (t, J = 7.1 Hz, 2H, Py-**H^3,5^**), δ 1.75 (s, 15H, Cp*-**H^methyl^**). Deuteration of Cp* is visible and highlighted in the spectrum in Figure S9. A small amount (<5%) of dissociated complex is visible in equilibrium with the complex, indicating the low stability of the mixed-ligand complex even in methanol.

^13^C NMR (CD_3_OD, ppm, 125 MHz, standard: δ 49.00 (solvent residual peak)): δ 153.91 (s, phen-**C^2,9^**), δ 152.59 (s, Py-**C^2,6^**), δ 146.89 (s, phen-**C^4a,6a^**), δ 141.88 (s, phen-**C^4,7^**), δ 141.79 (s, Py-**C^4^**), δ 132.63 (s, phen-**C^10a,10b^**), δ 129.48 (s, phen-**C^3,8^**), δ 129.25 (s, phen-**C^5,6^**), δ 129.04 (s, Py-**C^3,5^**), δ 121.79 (q, J_13C–19F_ = 318.5 Hz, triflate-**CF_3_**), δ 100.93 (d, J_13C–103Rh_ = 7.3 Hz, Cp*-**C^ring^**), δ 8.20 (s, Cp*-**C^-CHD2^**). High degree of deuteration of Cp* is visible and highlighted in the spectrum in Figure S10.

**Complex 5**: [RhCp*(8HQH_−1_)Cl] (27.2 mg, 65 μmol) and 1-methylimidazole (51.8 μL, 650 μmol); yield: 10.5 mg (21%). Elemental analysis calculated for C_47_H_47_BN_3_ORh: C 72.04%, H 6.05%, N 5.36%, O 2.04%. Found: C 72.09%, H 6.10%, N 5.43%, O 2.13%.

**Acetone-d_6_** was used as solvent, since in methanol the salt has insufficient solubility.

^1^H NMR (acetone-d_6_, ppm, 500 MHz, standard: δ 2.05 (solvent residual peak)): δ 9.09 (d, J = 4.8 Hz, 1H, 8HQ-**H^2^**), δ 8.31 (d, J = 8.4 Hz, 1H, 8HQ-**H^4^**), δ 7.94 (s, 1H, mim-**H^2^**), δ 7.60 (dd, J = 8.2 Hz, 4.9 Hz, 1H, 8HQ-**H^6^**), δ 7.34 (s, 8+1H, BPh_4_^−^-**H^2,6^** & 8HQ-**H^3^**), δ 7.14 (s, 1H, mim-**H^4^**), δ 7.07 (s, 1H, mim-**H^5^**), δ 6.92 (t, J = 7.1 Hz, 8+2H, BPh_4_^−^-**H^3,5^**&8HQ-**H^5^** and **H^7^**), δ 6.77 (t, J = 7.1 Hz, 4H, BPh_4_^−^-**H^4^**), δ 3.69 (s, 3H, mim-**H^methyl^**), δ 1.74 (s, 15H, Cp*-**H^methyl^**).

^13^C NMR (acetone-d_6_, ppm, 125 MHz, standard: δ 29.84 (solvent residual peak)): δ 165.01 (q, J_13C–11B_ = 98.7 Hz, BPh_4_^−^-**C^1^**), δ 148.48 (s, 8HQ-**C^2^**), δ 139.36 (s, 8HQ-**C^4^**), δ 137.07 (s, BPh_4_^−^-**C^2,6^**), δ 131.70 (s, 8HQ-**C^4a^**), δ 131.29 (s, 8HQ-**C^3^**), δ 128.59 (s, mim-**C^4^**), δ 125.99 (q, J_13C–11B_ = 2.5 Hz, BPh_4_^−^-**C^3,5^**), δ 123.81 (s, 8HQ-**C^6^**), δ 123.64 (s, mim-**C^5^**), δ 122.22 (s, BPh_4_^−^-**C^4^**), δ 115.41 (s, 8HQ-**C^5^**), δ 111.52 (s, 8HQ-**C^7^**), δ 95.85 (d, J_13C–103Rh_ = 8.1 Hz, Cp*-**C^ring^**), δ 34.87 (s, mim-**C^methyl^**), δ 8.66 (s, Cp*-**C^methyl^**). Coupling with ^11^B is highlighted in Figure S12.

**Complex 6**: [RhCp*(8HQH_−1_)Cl] (27.2 mg, 65 μmol) and pyridine (52.6 μL, 650 μmol); yield: 15.5 mg (31%). Elemental analysis calculated for C_48_H_46_BN_2_ORh: C 73.85%, H 5.94%, N 3.59%, O 2.05%. Found: C 73.80%, H 5.99%, N 3.68%, O 2.14%.

**Acetone-d_6_** was used as solvent since in methanol the salt has insufficient solubility.

^1^H NMR (acetone-d_6_, ppm, 500 MHz, standard: δ 2.05 (solvent residual peak)): δ 9.24 (d, J = 4.8 Hz, 1H, 8HQ-**H^2^**), δ 8.76 (d, J = 5.4 Hz, 2H, Py-**H^2,6^**), δ 8.35 (d, J = 8.4 Hz, 1H, 8HQ-**H^4^**), δ 7.97 (t, J = 7.4 Hz, 1H, Py-**H^4^**), δ 7.67 (dd, J = 8.1 Hz, 4.9 Hz, 8HQ-**H^6^**), δ 7.54 (t, J = 6.6 Hz, 2H, Py-**H^3,5^**), δ 7.34 (s, 8+1H, BPh_4_^−^-**H^2,6^**&8HQ-**H^3^**), δ 6.97 (d, J = 7.8 Hz, 1H, 8HQ-**H^5^**), δ 6.92 (t, J = 7.1 Hz, 8+1H, BPh_4_^−^-**H^3,5^** & 8HQ-**H^7^**), δ 6.77 (t, J = 7.1 Hz, 4H, BPh_4_^−^-**H^4^**), δ 1.75 (s, 15H, Cp*-**H^methyl^**).

^13^C NMR (acetone-d_6_, ppm, 125 MHz, standard: δ 29.84 (solvent residual peak)): δ 165.01 (q, J_13C–11B_ = 98.7 Hz, BPh_4_^−^-**C^1^**), δ 152.24 (s, Py-**C^2,6^**), δ 148.65 (s, 8HQ-**C^2^**), δ 140.61 (s, Py-**C^3,5^**), δ 139.81 (s, 8HQ-**C^4^**), δ 137.07 (s, BPh_4_^−^-**C^2,6^**), δ 131.38 (s, 8HQ-**C^3^**), δ 127.67 (s, Py-**C^4^**), δ 125.99 (q, J_13C–11B_ = 2.5 Hz, BPh_4_^−^-**C^3,5^**), δ 124.11 (s, 8HQ-**C^6^**), δ 122.22 (s, BPh_4_^−^-**C^4^**), δ 115.61 (s, 8HQ-**C^5^**), δ 112.11 (s, 8HQ-**C^7^**), δ 96.43 (d, J_13C–103Rh_ = 8.4 Hz, Cp*-**C^ring^**), δ 8.58 (s, Cp*-**C^methyl^**). Coupling with ^11^B is highlighted in Figure S14.

#### 2.2.2. Characterization Methods

Elemental analyses for complexes **1–6** were performed by the Microanalytical Laboratory of the University of Vienna on an EA3000 CHNSO analyzer, manufactured by Eurovector (Pavia, Italy). Samples were weighed on a Sartorius SEC 2 ultra-micro balance (Sartorius AG, Göttingen, Germany) with ±0.1 µg resolution. Sample weights from 1–3 mg were used. For calibration, two NIST-certified reference materials were used: sulfanilamide (C_6_H_8_N_2_O_2_S) and BBOT (C_26_H_26_N_2_O_2_S). The limit of quantification (LOQ) was 0.05% (*w*/*w*) for C, H, and N and 0.02% (*w*/*w*) for S. Additionally, for samples without N and/or S, the content of these elements was determined and verified to be below LOQ. The presented values are the average of the determinations in triplicate. NMR spectroscopic studies were carried out on a Bruker Avance III HD Ascend 500 Plus instrument (Billerica, MA, USA). Solid complexes were dissolved in CD_3_OD or in acetone-*d_6_*, and the solvent residual peaks were used for reference [32]. ^1^H NMR, ^13^C attached proton test (APT) NMR, and, in some cases, 2D NMR techniques (HMBC, HSQC, and COSY) were used to assign all peaks in the spectra. Characterization also included structural determination based on single-crystal X-ray diffractometry. Suitable crystals were measured with Bruker X8 APEX II, Bruker D8, or Stoe Stadivari diffractometer (STOE & Cie GmbH, Darmstadt, Germany) equipped with multilayer monochromator, Mo K/α, and Cu K/α INCOATEC and AXO micro focus sealed tubes (Incoatec GmbH, Geesthacht, Germany and AXO Dresden GmbH, Dresden, Germany) and Oxford cooling devices (Oxford Cryosystems Ltd., Oxford, United Kingdom). The structures were solved by direct and iterative Methods. Non-hydrogen atoms were refined with anisotropic displacement parameters. Hydrogen atoms were inserted at calculated positions and refined with a riding model. The following software was used: Bruker SAINT software package (Bruker SAINT v8.38B & v8.37A, Bruker AXS, 2005–2019), using a narrow-frame algorithm for frame integration, SADABS (Sheldrick, G.M.: SADABS, University of Göttingen: Niedersachsen, Germany, 1996) for absorption correction, X-Area Integrate Software package (X-Area Integrate 2.5.3.0, STOE, 2021), X-Area LANA for scaling (X-Area LANA 2.7.5.0, STOE, 2022), OLEX2 [33] for structure solution, refinement, molecular diagrams, and graphical user-interface, ShelXle [34] for refinement and graphical user-interface, SHELXS-2015 (Sheldrick, G.M.: SHELXS v2016/4, University of Göttingen: Niedersachsen, Germany, 2015) for structure solution, SHELXL-2015 (Sheldrick, G.M.: SHELXL v2016/4, University of Göttingen: Niedersachsen, Germany, 2015) for refinement, and Platon [35] for symmetry check were used. Information about data quality can be found in Figures S15–S20. The crystallographic data files for complexes **1′-6′** have been deposited with the Cambridge Crystallographic Database as CCDC 2209815–2209820.

### 2.3. pH-Potentiometry

pH-potentiometric measurements were performed to determine the protonation constants (***K***_p_) of the ligands and the concentration of the stock solutions. These measurements were carried out at 25.0 ± 0.1 °C in water in a pH range between 2.0 and 11.5, titrating with carbonate-free KOH solution (0.10 M). Samples had a constant ionic strength (*I* = 0.10 M (KCl)), and the water ionization constant (p***K***_w_) was determined as 13.76 ± 0.01 [36]. A Thermo Scientific 0710A0 benchtop pH meter (Waltham, MA, USA), equipped with a Metrohm combined electrode (type 6.0234.100) and a Metrohm 665 Dosimat burette (Metrohm, Herisau, Switzerland) was used for these measurements. The electrode system was calibrated, as suggested by Irving et al. [37]. The initial volume of the samples was 10.0 mL, and the ligand concentration was 2.0 mM in the samples. Samples were degassed by bubbling purified argon prior to and during the measurements. The computer program Hyperquad2013 [28] was utilized to determine the protonation constants of the ligands and concentration.

### 2.4. UV-Visible and NMR Spectroscopy

An Agilent Cary 8454 diode array spectrophotometer (Agilent Technologies, Santa Clara, CA, USA) was used to record the UV-visible (UV-vis) spectra in the 200–800 nm wavelength range. The path length was varied between 0.2 and 1 cm. *K*_p_ of benzimidazole and *K*_a_ of the aqua complexes were determined by spectrophotometric titrations (*I* = 0.1 M (KCl)). The mixed-ligand complex formation was investigated in two ways: (i) the kinetics of complex formation was studied in a given complex–to–monodentate ligand ratio (1:2 or 1:10) and followed by time; (ii) in a series of samples, the complex–to–monodentate ligand ratio was varied (0–10 equivalents), and after the given waiting time, the spectra were recorded. In a typical measurement, samples contained 200 μM complex, and PBS’ buffer was used to keep the pH = 6.0 or 7.4.

NMR spectroscopic studies were carried out on a Bruker Avance III HD Ascend 500 Plus instrument. For the aqueous samples, ^1^H NMR spectra were recorded with the WATERGATE water suppression pulse scheme using DSS internal standard. The highest peak of DSS was 0.00 ppm. The measurement was very similar to the UV-vis measurement since the complex–to–monodentate ligand ratio was also between 0–10 equivalents; the spectra were recorded after the given waiting time. Samples had a 10% (*v*/*v*) D_2_O content and had higher concentrations of the complexes (300 μM) than the photometric samples. This technique was useful in those cases where no spectral change was visible during the UV-vis titrations.

In both measurement methods, the formation constant (*K*) was obtained using the computer program PSEQUAD [38].

### 2.5. In Vitro Studies: Anticancer Assays

#### 2.5.1. Cell Lines and Culture Conditions

All cell culture reagents were obtained from Sigma-Aldrich (Merck KGaA, Darmstadt, Germany) and plasticware from Sarstedt (Nümbrecht, Germany). Human colonic adenocarcinoma cell lines Colo205 (catalog number: ATCC-CCL-222) and its doxorubicin-resistant counterpart Colo320 (catalog number: ATCC-CCL-220.1) were purchased from LGC Promochem (Teddington, UK). The cells were cultured in RPMI 1640 medium supplemented with 10% heat-inactivated fetal bovine serum, 2 mM *L*-glutamine, 1 mM sodium pyruvate, and 100 mM HEPES buffer, adjusting the pH to 6.0 or 7.0. The cells were incubated at 37 °C in a 5% CO_2_–95% air atmosphere. The semiadherent human colon cancer cells were detached with Trypsin-Versene solution for 5 min at 37 °C.

#### 2.5.2. MTT Assay

The solutions were freshly prepared prior to measurements (see Section 2.1). Due to the limited water solubility of econazole, the samples contained 50% (*v*/*v*) ethanol, which did not have an effect on cell viability at the final concentration (<1% ethanol). Samples without econazole also contained the same amount of ethanol for proper comparison. Firstly, these samples were diluted in complete culture medium; then, two-fold serial dilutions of the compounds were prepared in 100 μL of RPMI 1640, horizontally. The semiadherent colonic adenocarcinoma cells were treated with Trypsin-Versene solution. They were adjusted to a density of 1 × 10^4^ cells in 100 μL of RPMI 1640 medium and were added to each well, with the exception of the medium control wells. The final volume of the wells containing compounds and cells was 200 μL. Samples were triplicated. The pH of the medium was adjusted to 6.0 or 7.0.

The culture plates were incubated at 37 °C for 72 h; at the end of the incubation period, 20 μL of MTT solution (from a stock solution of 5 mg/mL) was added to each well. After incubation at 37 °C for 4 h, 100 μL of SDS solution (10% in 0.01 M HCI) was added to each well, and the plates were further incubated at 37 °C overnight. Cell growth was determined by measuring the optical density (OD) at 540/630 nm with a Multiscan EX ELISA reader (Thermo Labsystems, Cheshire, WA, USA). Inhibition of cell growth (expressed as IC_50_: the inhibitory concentration that reduces by 50% the growth of the cells exposed to the tested compounds) was determined from the sigmoid curve where:

inhibition = 100 − ((OD_sample_ − OD_medium control_)/(OD_cell control_ − OD_medium control_)) × 100

values were plotted against the logarithm of compound concentrations. Curves were fitted using GraphPad Prism software (2022, version 9.4.1, Graphpad Software (LLC) d.b.a Dotmatics, San Diego, CA, USA) using the sigmoidal dose–response model (comparing variable and fixed slopes).

### 2.6. In Vitro Studies: Antibacterial Assays

The antibacterial effect was studied in two Gram-positive and in two Gram-negative strains. The Gram-positive *S. aureus* ATCC 25923 (ATCC, Manassas, VA, USA) is a methicillin-susceptible reference bacterial strain, while *S. aureus* 272123 is a clinical isolate and resistant to methicillin and ofloxacin (MRSA), which was given by Prof. Dr. Leonard Amaral (Institute of Hygiene and Tropical Medicine, Lisbon, Portugal). The Gram-negative strains were *E. coli* ATCC 25922 (ATCC, Manassas, VA, USA), which is sensitive to antibiotics, and *E. coli* 32313 (provided by the Institute of Clinical Microbiology, University of Szeged, Hungary), which is resistant to different antibiotics (trimethoprim/sulfamethoxazole, gentamicin, ciprofloxacin, and norfloxacin).

The minimum inhibitory concentrations (MICs) of the compounds were determined in 96-well plates based on the Clinical and Laboratory Standard Institute guidelines [39]. The compounds were diluted in 100 μL of Mueller Hinton broth. Then, an overnight bacterial culture diluted 10,000 times in 100 μL of medium was added to each well, with the exception of the medium control wells. For each strain and each tested compound, four pH values were set (5, 6, 7, and 8). Each sample was triplicated. The plates were further incubated at 37 °C for 18 h, and at the end of the incubation period, the MIC values of the tested compounds were determined by visual inspection.

## 3. Results and Discussion

### 3.1. Selected Complexes and Ligands

In an ideal acidosis-activated mixed-ligand complex, the bidentate ligand is bound to the metal center with such high stability that its dissociation should not take place at pH 7.4, which is the pH of the blood. Based on their anticancer effects and high stability within a wide pH range [19,21], the complexes of ethylenediamine, 1,10-phenanthroline, and 8-hydroxyquinoline formed with [RhCp*(H_2_O)_3_]^2+^ and [Ru(*p*-cym)(H_2_O)_3_]^2+^ organometallic cations were chosen for our studies (Chart 2) as the core complexes (aqua complexes, [M(arene)(N,N/O)(H_2_O)]^2+/+^). Notably, the Ru(*p*-cym) complexes were used for comparisons in some studies.

There are examples in earlier studies for the mixed-ligand complexes of monodentate ligands with an oxygen donor atom (general structure: [M(arene)(X,Y)(O-donor)]), in which the complexes release the monodentate ligand rapidly (minutes after dissolution in water); thus, the activation step takes place prior to reaching the target tissue [40,41,42]. In contrast, the presence of phosphorus and sulfur donor atoms in the monodentate ligands results in mixed-ligand complexes with outstanding stability, as was already reported for RuCp complexes containing PPh_3_ and for RAPTA complexes (vide supra) [12,18]. The liberation of these monodentate ligands is not feasible at pH 7.4. Between these two extremes, the co-ordination ability of monodentate ligands with a nitrogen donor atom seems to be an optimal choice. The variability of nitrogen-containing functional groups gives an opportunity to optimize the monodentate ligands in the mixed-ligand complexes. In this work, small molecules bearing a nitrogen donor atom (methylamine, benzylamine, 1-methylimidazole, and pyridine in Chart 2) were chosen, for which the structures are frequently occurring motifs in drug molecules. The binding of these ligands to half-sandwich [M(arene)(N,N/O)(H_2_O)]^2+/+^ aqua complexes in an aqueous solution, their anticancer effects, and the synthesis of the six chosen complexes will be introduced in the next chapters.

### 3.2. Effect of pH on the Stability of the Mixed-Ligand Complexes: Screening for Monodentate Ligands

**Figure 1:**
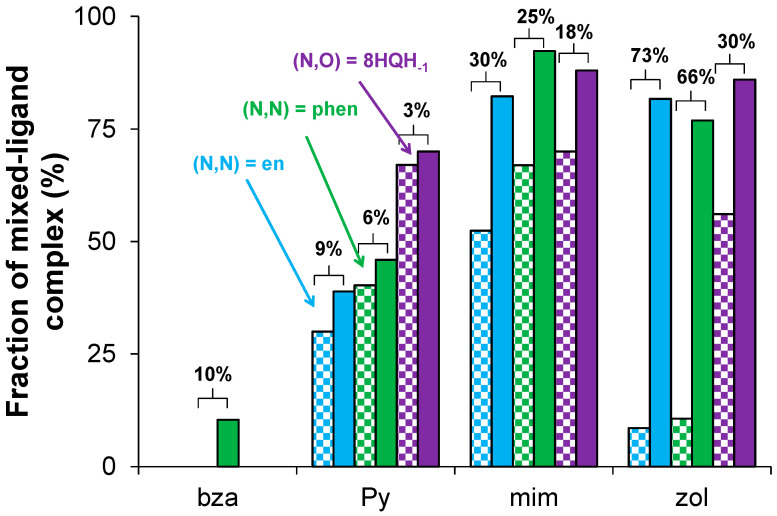
Fractions of the formed mixed-ligand [RhCp*(N,N/O)(N)]^2+/+^ complexes at *c*(complex):*c*(monodentate ligand) = 1:1 ratio measured at pH = 6.0 (full bars) and 7.4 (checkered bars). Fractions were determined by the integration of the peaks in the ^1^H NMR spectra. The percentage values above the columns show the difference in the fractions of the mixed-ligand complex at the two pH values. {*c*([RhCp*(phen)(H_2_O)]^2+^) = *c*([RhCp*(en)(H_2_O)]^2+^) = 1 mM; *c*([RhCp*(8HQH_−1_)(H_2_O)]^+^) = 500 μM; 10% D_2_O; pH = 6.0 or 7.4 (PBS’); *t* = 25.0 °C}.

For this study, [M(arene)(N,N/O)(H_2_O)]^2+/+^ complexes were chosen, in which the rupture of the co-ordination bond between the metal center and the bidentate ligand is not expected in the pH range of 5–8 due to their considerably high stability constants (see Section 3.1) [19,21]. In the preliminary studies, the co-ordination ability of the monodentate ligands to the [RhCp*(en)(H_2_O)]^2+^, [RhCp*(phen)(H_2_O)]^2+^, and [RhCp*(8HQH_-1_)(H_2_O)]^+^ complexes was tested (Chart 2). The samples contained the aqua complex and the monodentate ligand in a 1:1 ratio and were buffered at pH = 6.0 and pH = 7.4. For an ideal drug candidate, all of the monodentate ligand would be bound at pH 7.4; thus, the mixed-ligand complex would be present exclusively, which dissociates completely at pH 6.0. The fractions (expressed as percentages in Figure 1) show that these complexes are far from the idealistic case; however, the mixed-ligand complexes were formed with different fractions at pH = 6.0 and 7.4. In the case of methylamine, no interaction was found under these conditions, whereas the binding of pyridine, 1-methylimidazole, and benzylamine was observed (Figure 1). A big difference emerged between the co-ordination of the heterocyclic nitrogen donors (Py, mim) and the aliphatic amines (bza, mea), as the latter ones form a minimal amount of or no mixed-ligand complexes, while the fraction of the complexes of mim were above 75% at pH 7.4. When comparing the complexes of bza, Py, mea, and mim, it could be concluded that the highest amount of monodentate ligand is liberated from the complexes of 1-methylimidazole, e.g., the fraction of [RhCp*(en)(mim)]^2+^ decreases from 82% to 52% under the given conditions (Figure 1).

**Scheme 2:**
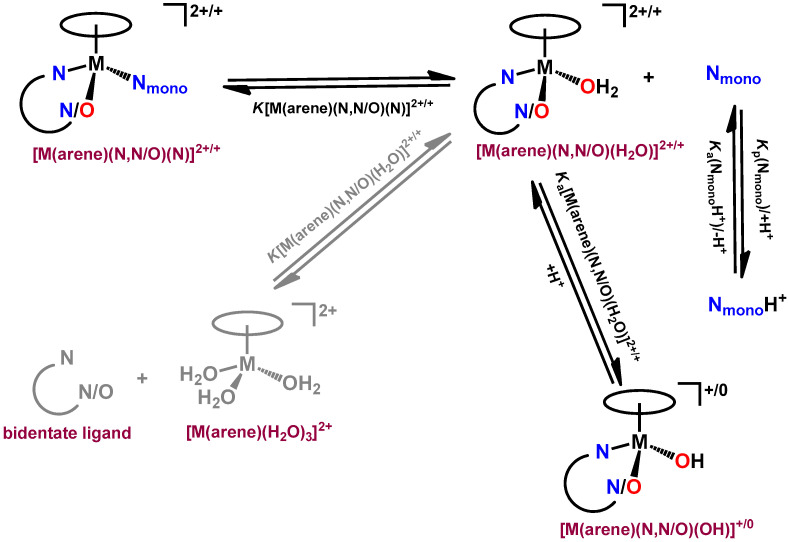
Coupled equilibrium processes of the title complexes in aqueous solution regarding the liberation of the monodentate N donor ligands from the mixed-ligand complexes ([M(arene)(N,N/O)(N)]^2+/+^), the liberation of bidentate ligand from [M(arene)(N,N/O)(H_2_O)]^2+/+^, the deprotonation of the co-ordinated aqua ligand (i.e., formation of the mixed hydroxido complex ([M(arene)(N,N/O)(OH)]^+/0^)), and protonation of the free monodentate ligand.

Aliphatic amines and aromatic nitrogen have different co-ordination abilities, and the heterocyclic compounds also show discrepancies from each other. The reason for the differences may be connected to their different protonation constant (*K_p_*), which were determined and are collected in Table 1. The protonation ability of the monodentate ligand has an impact on its liberation with decreasing pH via the coupled equilibria, as Scheme 2 shows. Additionally, the deprotonation of the aqua complex of the bidentate ligand is also possible by increasing pH (Scheme 2). Therefore, the characterization of the coupled (de)protonation equilibrium processes is also needed to better understand the apparent stability of the mixed-ligand complexes at various pH values.

The selected monodentate ligands are involved only in one protonation equilibrium process in the investigated pH range (pH = 2–11.5). The protonation constants (*K_p_*) were determined by pH-potentiometric and UV-vis spectrophotometric titrations in the presence of 0.1 M KCl (see Table 1 and Figures S21 and S22 for examples). Notably, the logarithm of the protonation constant (log*K*_p_) of these ligands is equal to the negative logarithm of the proton dissociation constant (p*K*_a_) of the protonated forms. The chloride ion concentration was selected according to the blood serum. As Table 1 shows, the log*K*_p_ values of the chosen monodentate ligands cover a wide range (5.32–10.58).

The proton dissociation of the aqua ligand in the [M(arene)(N,N/O)(H_2_O)]^2+/+^ complexes (Scheme 2) results in the inert mixed-hydroxido species (general formula: [M(arene)(N,N/O)(OH)]^+/0^). The corresponding p*K*_a_ values were also determined under used conditions and are collected in Table S1. Based on those values, the mixed-hydroxido complexes appear only in an insignificant amount (less than 3%) at pH 7.4 (as p*K*_a_ ≥ 8.9); thus, the influence of this process on the apparent stability of the mixed-ligand complexes is negligible.

Based on these preliminary studies, the optimal monodentate ligand has a similar or higher log*K*_p_ than 5.32 and much less than 9.42. Besides the log*K*_p_, the complex-formation ability should be sufficient at pH 7.4. While the first criterion can be tuned by functionalization, the second one depends on the type of the nitrogen donor atom, and imidazole-like nitrogen seems to form highly stable complexes. In order to prove this assumption, zoledronic acid was also tested as a potential monodentate ligand, which has a similar binding moiety as 1-methylimidazole (Chart 2). It is a bisphosphonate used against bone fractions in bone cancer [48], having three overlapping log*K*_p_ values (8.13, 7.48, and 6.67) predicted by the Marvin program (version 18.23.0; ChemAxon: Budapest, Hungary, 2018). As shown in Figure 1, this ligand liberates from its metal complexes to an even greater extent than 1-methylimidazole when the pH drops from 7.4 to 6.0. Although its complexes are nice examples of how functionalization can improve the acid-sensitivity of mixed-ligand complexes, they were not used in further experiments due to the lack of anticancer and antibacterial effects of zoledronic acid.

Beside zoledronic acid and two other imidazole derivatives were also chosen in this work (Chart 2). Econazole is an antimycotic compound having anticancer activity [49]. Due to the low aqueous solubility of econazole, it was only used for the biological assays. Benzimidazole is a frequently occurring structural motif in drug molecules, e.g., in proton pump inhibitors (pantoprazole, etc.) [6]. The coadministration of benzimidazole-containing inhibitors with anticancer drugs can improve the therapeutic effect; moreover, some of them are able to inhibit cell proliferation and induce apoptosis in cancer cells [6]. Benzimidazole is also of interest since it combines the imidazole-like nitrogen donor atom; however, its protonation constant is lower than that of 1-methylimidazole (Table 1).

### 3.3. Stability of the Mixed-Ligand Complexes

The selection of the most promising monodentate ligands (heterocyclic compounds: Py, mim, and bim) was followed by the determination of the equilibrium constants for the formation of their mixed-ligand RhCp* complexes. For comparative purposes, the formation constants of analogous half-sandwich Ru(*p*-cym) complexes were determined as well.

**Figure 2:**
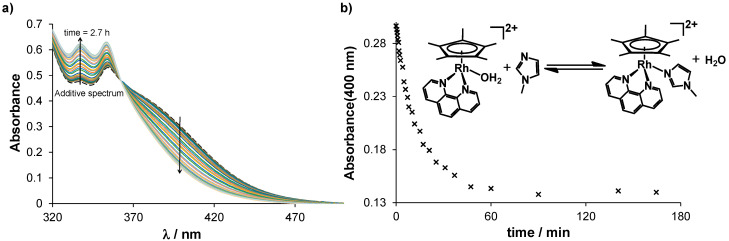
(**a**) Time-dependent UV-vis spectra of the *c*([RhCp*(phen)(H_2_O)]^2+^):*c*(1-methylimidazole) = 1:2 mixture under physiological conditions (pH = 7.4 (PBS’ buffer)). (**b**) Time dependence of the absorbance values at 400 nm. {*c*([RhCp*(phen)(H_2_O)]^2+^) = 200 μM; *c*(1-methylimidazole) = 400 μM; pH = 7.40 (PBS’); *t* = 25 °C; *ℓ* = 1 cm}.

Firstly, the reactions were followed by UV-vis spectrometry, and significant changes in the charge transfer bands were observed. Based on these preliminary measurements, the waiting time needed prior to measurement was determined. Generally, the reaction was faster for the RhCp* complexes than for the Ru(*p*-cym) analogs. The 8-hydroxyquinoline complexes showed the fastest reaction in both cases. The [Ru(*p*-cym)(en/phen)(H_2_O)]^2+^ complexes reached chemical equilibrium within only hours, while the reaction between 1-methylimidazole and [RhCp*(phen)(H_2_O)]^2+^ (Figure 2) or [RhCp*(en)(H_2_O)]^2+^ (Figure S23) was completed within 90 min. However, the fractions of the mixed-ligand complexes cannot be determined from these spectral changes in the chemical equilibrium; thus, further experiments were carried out to determine the formation constants.

The rate of the release of the monodentate ligand from the synthesized mixed-ligand complex is also of interest. Although a mixed-ligand complex with low stability should release its monodentate ligand easily, kinetic inertness has a major effect on reaching the equilibrium state. In contrast to their non-organometallic octahedral counterparts, half-sandwich Ru(II) and Rh(III) complexes have faster ligand exchange reactions [18]. The release of the pyridine ligand from the synthesized complex 2 was followed by ^1^H NMR spectroscopy (Figure S24a). While ~75% of the monodentate ligand dissociated at pH = 6.0 (Figure S24b) after 5 min, chemical equilibrium was reached with ~94% released monodentate ligand only after half an hour, and similar behavior was found at pH = 7.4. These measurements prove that the equilibrium state can be reached within minutes/hours, including both the formation and dissociation of the mixed-ligand complexes.

**Figure 3:**
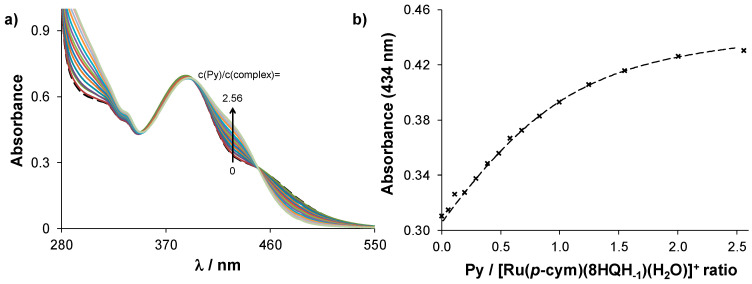
(**a**) UV-vis spectra of *c*([Ru(*p*-cym)(8HQH_-1_)(H_2_O)]^+^) at various pyridine concentrations at pH = 5.95 (PBS’ buffer)). (**b**) Absorbance values at 434 nm as the function of *c*(Py)/*c*(aqua complex) ratio. {*c*([Ru(*p*-cym)(8HQH_-1_)(H_2_O)]^+^) = 200 μM; pyridine: 0–2.56 equivalents; pH = 5.95 (PBS’); *t* = 25 °C; *ℓ* = 1 cm}.

**Figure 4:**
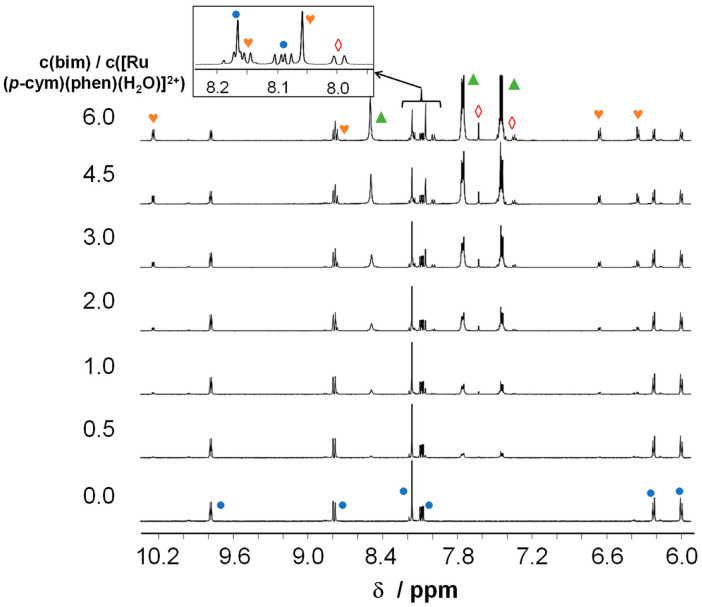
Low-field region of ^1^H NMR spectra of ([Ru(*p*-cym)(phen)(H_2_O)]^2+^ at various benzimidazole/complex ratios. Peak assignation: [Ru(*p*-cym)(phen)(H_2_O)]^2+^: ●; free benzimidazole: ▲; [Ru(*p*-cym)(phen)(bim)]^2+^: ♥; co-ordinated benzimidazole: **◊**. {*c*([Ru(*p*-cym)(phen)(H_2_O)]^2+^) = 300 μM; pH = 5.95 (PBS‘); 10% D_2_O; *t* = 25.0 °C}.

Formation constants for the formation of the mixed-ligand complexes (log*K*([M(arene)(N,N/O)(N)]) were determined at pH = 6.0 and at pH = 7.4 by UV-vis spectrophotometry and ^1^H NMR spectroscopy. Batch samples were prepared, and after the appropriate waiting time (based on the preliminary kinetic studies), the spectra were recorded. A clear spectral change is visible in the UV-vis spectrum of [Ru(*p*-cym)(8HQH_−1_)(H_2_O)]^+^, when pyridine is added gradually to the complex (Figure 3). When the spectral change was not suitable for calculation (ΔA < 0.1), the ^1^H NMR spectra were recorded. Figure 4 shows an example of a series of ^1^H NMR spectra recorded for the [Ru(*p*-cym)(phen)(H_2_O)]^2+^ complex with increasing benzimidazole concentration.

**Figure 5:**
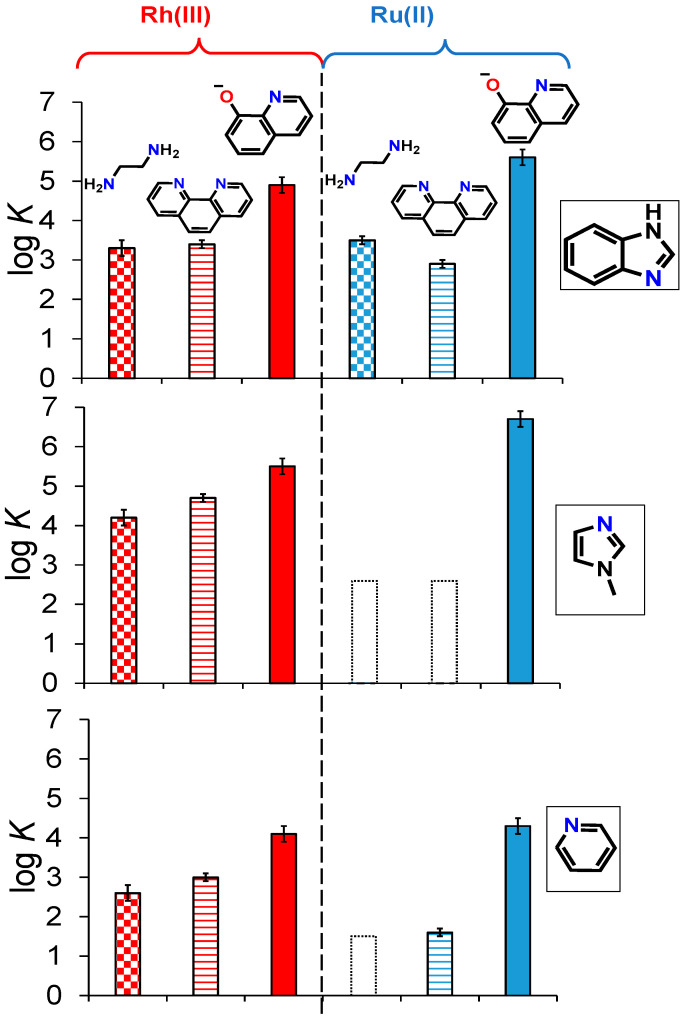
Formation constants (log*K*) determined for mixed-ligand half-sandwich complexes of Rh(III) and Ru(II) by UV-vis and ^1^H NMR spectroscopy. Empty bars with a dotted line indicate maximum values, which could not be determined under the given conditions. Values are collected in Table S2. {*I* = 0.10 M (KCl); *t* = 25.0 °C}.

It is worthwhile to mention that the liberation of the bidentate ligand was not detected even at higher monodentate ligand excess (~10), which means that the disruption of the bond between the bidentate ligand and the metal center is not feasible under these conditions. The calculated formation constants are compared in Figure 5 and are collected in Table S2. The formation of the mixed-ligand complexes was not detected in three cases under the applied conditions, namely [Ru(*p*-cym)(en)(mim)]^2+^, [Ru(*p*-cym)(phen)(mim)]^2+^, and [Ru(*p*-cym)(en)(Py)]^2+^. For these complexes, only a higher limit of the formation constant could be given, assuming that a maximum of 5% of the complex would form (limit of detection) under the conditions used for ^1^H NMR spectroscopy. Based on Figure 5, the following trends are observed in the formation constants: (i) [RhCp*(en)(H_2_O)]^2+^ and [RhCp*(phen)(H_2_O)]^2+^ have a stronger monodentate ligand-binding ability than the Ru(*p*-cym) analogs; (ii) the formation constants of the [Ru(*p*-cym)(8HQH_-1_)(N)]^+^ complexes are similar or somewhat higher than those of the RhCp* analogs; (iii) a stability trend for the monodentate ligand: mim > bim > Py (iv) and the bidentate ligand: 8HQH_-1_ > phen ≥ en can be seen.

**Figure 6:**
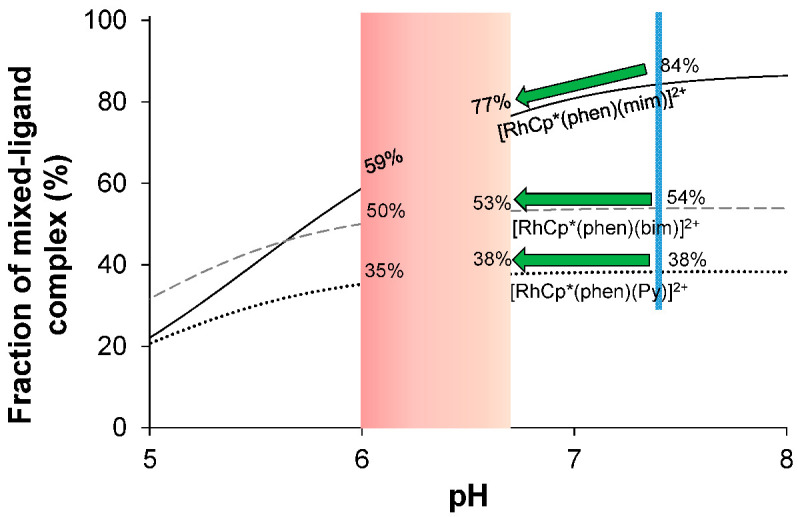
Fractions of the mixed-ligand complexes of [RhCp*(phen)(H_2_O)]^2+^ formed with pyridine, benzimidazole, and 1-methylimidazole as a function of pH. Formation constants shown in Figure 5 and in Table S2 were used for the calculation. Percentages indicate the fraction at pH = 7.4 (blue line) and in the range between pH = 6.0–6.7 (pink rectangle: possible pH of tumor microenvironment during acidosis).

With the help of these formation constants, the extent of complex formation can be estimated for a chosen monodentate ligand—complex chemical system under the desired conditions (concentration and pH). Figure 6 and Figure S25 show the computed fractions of the formed, mixed-ligand complexes between pH = 5 and 8 and at a 100 μM concentration (metal complex-to-monodentate ligand ratio = 1:1). The fraction at pH = 7.4 and at pH 6.0–6.7 were compared to each other. The complex with greater acid-sensitivity has a higher difference in percentage. For instance, when the pH decreases from 7.4 to 6.0, 25% of [RhCp*(phen)(mim)]^2+^ dissociated, while the benzimidazole and pyridine analogs showed negligible sensitivity (3–4% difference, Figure 6). Figure S25 shows that the difference is only 10% for [Ru(*p*-cym)(8HQH_−1_)(mim)]^+^ and 13% for [RhCp*(8HQH_−1_)(mim)]^+^ at decreased pH due to their higher stability.

Similar to the case of a decreased pH, the lower concentration also shifts the equilibrium in the direction of dissociation. As an example, the fractions of [Ru(p-cym)(8HQH_−1_)(mim)]^+^ are shown in Figure S26 with decreasing concentration. The fraction of the active form at pH = 6.0 is only 5% at a concentration of 1 mM, while it increases to 41% when the concentration drops by two orders of magnitude. When considering this finding, these compounds exhibit contradictory behavior since the sample with a lower concentration shows a higher fraction of the activated complex.

### 3.4. Structural Characterization of Mixed-Ligand Complexes

The mixed-ligand complexes of 1-methylimidazole show sufficient stability and acid-sensitivity as well, which is in contrast to the complexes of pyridine. It is clear that the acid–base properties of the monodentate ligand play a crucial role in the acid-sensitivity of the complexes; however, the structural comparison of the complexes may show certain trends as well.

For the previous studies, the mixed-ligand complexes were prepared in situ in the samples prior to the measurements (screening of the type of the monodentate ligand, formation constant determination, etc.). For the structural comparison, the six selected RhCp* complexes were synthesized (complexes **1–6**), and single crystals were grown using the solutions of these compounds. Since the crystals are different in their composition (solvent content and anion) from complexes **1–6**, they are abbreviated as complexes **1′–6′**.

The synthesis of the complexes is shown in Scheme 1 and is described in Section 2.2.1. After removing the chloride ions from the [RhCp*(en/phen)Cl](CF_3_SO_3_) and [RhCp*(8HQH_−1_)Cl] complexes, a 10-fold excess of the monodentate ligand (pyridine or 1-methylimidazole) was applied. The solution was concentrated, and the forming solid was filtered, recrystallized, and washed with ether and *n*-hexane. The single crystals were grown using the vapor diffusion and slow evaporation methods. Notably, tetraphenylborate anion induced crystal formation better than triflate anion since its solubility in methanol decreased. It also has a similar size compared to the cationic mixed-ligand complexes, which helps with crystallization. The crystal data, data collection parameters, and the structure refinement details for complexes **1′–6′** are given in Tables S3–S5.

**Figure 7:**
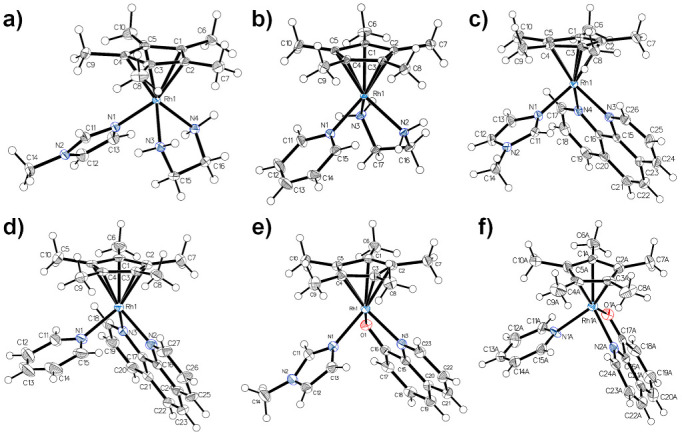
Molecular structure of (**a**) [RhCp*(en)(mim)](CF_3_SO_3_)_2_ (**1′**), (**b**) [RhCp*(en)(Py)](CF_3_SO_3_)_2_ (**2′**), (**c**) [RhCp*(phen)(mim)](BPh_4_)_2_×2 CH_2_Cl_2_ (**3′**), (**d**) [RhCp*(phen)(Py)](CF_3_SO_3_)_2_×CH_3_OH (**4′**), (**e**) [RhCp*(8HQH_−1_)(mim)](BPh_4_)×CH_3_OH (**5′**), and (**f**) [RhCp*(8HQH_-1_)(Py)](BPh_4_) (**6′**). Solvent molecules and counter ions are omitted for clarity. Displacement ellipsoids are drawn at a 50% probability level.

Three 1-methylimidazole and three pyridine complexes were crystallized, namely [RhCp*(en)(mim)](CF_3_SO_3_)_2_ (**1′**), [RhCp*(phen)(mim)](BPh_4_)_2_×2 CH_2_Cl_2_ (**2′**), [RhCp*(8HQH_−1_)(mim)](BPh_4_)×CH_3_OH (**3′**), [RhCp*(en)(Py)](CF_3_SO_3_)_2_ (**4′**), [RhCp*(phen)(Py)](CF_3_SO_3_)_2_×CH_3_OH (**5′**), and [RhCp*(8HQH_−1_)(Py)](BPh_4_) (**6′**). Single crystals that were capable of structural determination were formed in all cases. The structures show the general “piano-stool” structure (Figure 7).

When comparing the Rh–N_mono_ bond lengths in Table 2, it is clear that most of the complexes of 1-methylimidazole (**1′,3′,5′**) have shorter bonds than the pyridine analogs (**2′,4′,6′**). It was proven in our previous works that the longer bond lengths between the donor atoms and metal ion may indicate the lower aqueous stability of the complex [19,30]. To apply this observation in a broader scope, the M–N_mono_ bond lengths of mixed-ligand half-sandwich Rh(III), Ru(II), Os(II) and Ir(III) complexes were collected and compared in Figure S27 [50,51,52,53,54,55,56,57,58,59,60,61]. The following general trend is visible in the bond length: M–imidazole < M–bim < M–Py < M–NH_3_. This is consistent with the experienced order of the stability constants.

### 3.5. In Vitro Anticancer and Antibacterial Activity of the Studied Compounds

The pH-dependent solution speciation of the selected mixed-ligand complexes is an important feature in the hypothesized mechanism of action, and the modeling of acidosis is necessary for the in vitro anticancer assays. In order to find out the cytotoxicity difference based on the pH of the medium, cytotoxic activity was determined at pH 6 and 7 by MTT assay in two human colon adenocarcinoma cell lines (doxorubicin-sensitive Colo205 and doxorubicin-resistant Colo320). In these assays, the mixed-ligand complexes were studied with the general structure of [RhCp*(8HQH_−1_)(N)]Cl and [RhCp*(phen)(N)]Cl_2_, where (N) is pyridine, 1-methylimidazole, or econazole, respectively. The mixed-ligand complexes were prepared prior to the measurements, as was already described in Section 2.1. With this method, the effect of the different counter ions [29] could be avoided, and a more adequate comparison of the mixed-ligand complexes could be conducted.

The selected complexes, [RhCp*(8HQH_−1_)Cl] and [RhCp*(phen)Cl]Cl, showed remarkable anticancer activity in other cancer cell lines in previous studies [19,21]. Econazole and 1-methylimidazole have imidazole-like nitrogen donor atoms that co-ordinate more strongly to the metal center than pyridine. As was mentioned (*vide supra*), econazole alone has cytotoxic effects in cancer cells [49]. Table 3 shows the pH-dependent anticancer effects of the mixed-ligand complexes. [RhCp*(phen)Cl]Cl, 1-methylimidazole, and pyridine exhibited no anticancer activity in these cancer cell lines (IC_50_ > 100 μM). A change of the bidentate ligand to 8-hydroxyquinoline resulted in a significant improvement in the cytotoxic activity. The notable activation of acidosis was observed. Although the [RhCp*(phen)(H_2_O)]^2+^ complex, pyridine, and 1-methylimidazole did not have any cytotoxic effect separately, their combination exhibited a measurable cytotoxic effect in Colo320 cells at pH = 6.

It is also noteworthy that the combination of [RhCp*(8HQH_−1_)(H_2_O)]^+^ with econazole showed additive or synergistic effects, reaching a superb IC_50_ = ~1.8 μM at pH 6, while almost double this level was measured at a higher pH. This is proof that dual-targeting is feasible with the combination of a monodentate ligand (of anticancer activity) and a cytotoxic complex.

During the chemotherapeutic treatment of cancer, patients often suffer from fungal, bacterial, or viral infections due to their weaker immune system (e.g., decreased white blood cell number) [62]. Therefore, it is crucial to monitor the efficacy of future chemotherapeutic compounds against these infections as well. Since resistant bacteria are the most dangerous, in these experiments, the commonly appearing methicillin-resistant *Staphylococcus aureus* (MRSA) strain and a resistant *Escherichia coli* strain were covered next regarding the nonresistant cell lines. These measurements were also performed at different pH values (5, 6, 7, and 8) (Table 4, Tables S6 and S7). However, when applying pH 5 and pH 8, only minor or no changes were seen when compared to pH 6 and 7, respectively; thus, only pH 6 and 7 are included in Table 4. All data are presented in Tables S6 and S7.

Similar to anticancer activity, the [RhCp*(phen)Cl]Cl complex, 1-methylimidazole, and pyridine showed no antibacterial effect against any of the bacterial strains (MIC > 100 μM), while econazole and all the complexes of 8-hydroxyquinoline were more effective. Synergism was also visible in these results; although the Gram-negative strains are more resistant to the mentioned compounds alone, econazole potentiates the antibacterial effect of both the half-sandwich complexes.

## 4. Conclusions

In this work, novel mixed-ligand half-sandwich Rh complexes (general formula: [RhCp*(N,N/O)(N)]^2+/+^) were developed, and their solution equilibria and anticancer effects were studied. The binding ability of the different monodentate ligands bearing nitrogen donor atoms was compared via NMR spectroscopy. The co-ordination of the heterocyclic compounds occurred to a greater extent than in the case of the amines. The stability constants for the formation of the [RhCp*(N,N/O)(N)]^2+/+^ and [Ru(*p*-cym)(N,N/O)(N)]^2+/+^ complexes in the reaction between the [RhCp*(N,N/O)(H_2_O]^2+/+^ and the monodentate ligands showed the following trends: (i) at pH 7.4, the formation constants were higher for the Rh(III) complexes than for the Ru(II) compounds; (ii) the log*K*_p_ (or p*K*_a_) of the monodentate ligand is related to the stability of the mixed-ligand complex; (iii) the (N,O^−^) donor set of the bidentate ligand facilitates the co-ordination of the monodentate ligand when compared to the (N,N) donor bidentate ligands.

The structural comparison of complexes **1′–6′** also suggests differences in the binding of the monodentate ligands. Shorter bond lengths between the metal ions and the nitrogen atom of the monodentate ligands indicate a higher formation constant, which correlates well with the experienced stability in an aqueous solution. This theory was applied to a broader group of half-sandwich compounds, and a similar trend was found in all cases. The anticancer studies in the Colo205 and Colo320 cells showed the cytotoxic effects of the mixed-ligand complexes and the additive or synergistic effects of the econazole complexes. The complexes of 8-hydroxyquinoline were more cytotoxic in the cancer cell lines than those of 1,10-phenanthroline. There were also signs of activation at a decreased pH (in the case of [RhCp*(phen)(Py/mim)]^2+^), which proved the hypothesis of the work. The antimicrobial studies showed the efficacy of the econazole complexes and the [RhCp*(8HQH_−1_)(Py/mim/econ)]^+^ complexes against the MRSA strain. Moreover, the complexes of econazole demonstrated antibacterial activity against the Gram-negative strains, which is a great improvement compared to the inactivity of econazole and the aqua complexes of the bidentate ligands against these strains.

Based on our results, the further development of the mixed-ligand half-sandwich complexes is straightforward since using active monodentate ligands may increase the cytotoxicity and the selectivity of these compounds. If the aqua complex and the monodentate ligand exert their anticancer effect against different targets (e.g., inhibiting specific cancer-related enzymes), dual-targeting and synergism are possible. Besides the use of specific anticancer monodentate ligands, switching to other aqua complexes with different metal centers (Ir(III) or Os(II)) may also increase the cytotoxic activity and selectivity of mixed-ligand half-sandwich complexes.

## Figures and Tables

**Table 1:** Protonation constants of the monodentate ligands (proton dissociation constants of the protonated forms) {*I* = 0.10 M (KCl); *t* = 25.0 °C}.

Compound	log*K*_p_ (= p*K*_a_)	Method	Literature Data
pyridine	5.32 ± 0.01	pH-potentiometry	5.24 [43]
benzimidazole	5.44 ± 0.01	UV-vis	5.54 [44]
1-methylimidazole	7.14 ± 0.01	pH-potentiometry	7.11 ^1^ [45]
benzylamine	9.42 ± 0.01	pH-potentiometry	9.43 [46]
methylamine	10.58 ± 0.03	pH-potentiometry	10.51 [47]

^1^ *I* = 0.10 M (KNO_3_).

**Table 2:** Selected bond distances (Å) and angles (°) in complexes **1′–6′**. Values in parenthesis are the standard deviations.

	1′	2′	3′
Bond lengths (Å)			
Rh–N1	2.126 (2)	2.146 (1)	2.129 (2)
Rh–N2	2.136 (2)	2.122 (1)	2.119 (2)
Rh–N_mono_^1^	2.119 (2)	2.127 (1)	2.088 (2)
Rh–C_g_^2^	1.776	1.775	1.786
Angles (°)			
N1–Rh–N2	79.92 (9)	79.53 (4)	77.78 (8)
N1–Rh–N_mono_^1^	85.47 (9)	88.72 (4)	91.10 (8)
N2–Rh–N_mono_^1^	59.57 (9)	89.14 (4)	85.38 (8)
	**4′**	**5′**	**6′** ^3^
Bond lengths (Å)			
Rh–N1	2.116 (3)	2.109 (1)	2.101 (6)
Rh–N2/O	2.111 (3)	2.088 (1)	2.079 (5)
Rh–N_mono_^1^	2.118 (3)	2.089 (1)	2.090 (6)
Rh–C_g_^2^	1.792	1.773	1.777
Angles (°)			
N1–Rh–N2/O	77.64 (12)	78.96 (5)	79.6 (2)
N1–Rh–N_mono_^1^	88.30 (12)	85.10 (5)	85.2 (2)
N2/O–Rh–N_mono_^1^	88.29 (12)	85.98 (5)	85.9 (2)

^1^ N_mono_ = nitrogen donor atom of monodentate ligand. ^2^ C_g_ is the center of gravity of arene ring. ^3^ Average of two molecules in one unit cell.

**Table 3:** In vitro cytotoxicity expressed as IC_50_ values (μM) for the [RhCp*(phen)(N)]^2+^ and [RhCp*(8HQH_−1_)(N)]^+^ complexes ((N) = econ, mim, or Py) in Colo205 and Colo320 human colon adenocarcinoma cell lines and at two different pH values. {72 h incubation time}.

	IC_50_ (μM)			
	Colo205		Colo320	
pH=	**6**	**7**	**6**	**7**
mim	>100	>100	>100	>100
Py	>100	>100	>100	>100
econ	23.1 ± 2.1	18.2 ± 0.7	10.3 ± 1.5	11.4 ± 0.4
[RhCp*(phen)Cl]Cl	>100	>100	>100	>100
+mim	>100	>100	71.7 ± 3.3	>100
+Py	>100	>100	83.6 ± 4.1	>100
+econ	15.5 ± 0.7	8.6 ± 1.6	14.1 ± 1.5	12.5 ± 1.8
[RhCp*(8HQH_−1_)Cl]	8.1 ± 0.5	8.8 ± 0.4	6.1 ± 0.2	3.3 ± 0.1
+mim	7.0 ± 0.4	7.2 ± 0.2	6.0 ± 0.1	6.1 ± 0.2
+Py	7.2 ± 0.4	7.3 ± 0.3	6.2 ± 0.5	5.9 ± 0.1
+econ	1.8 ± 0.1	3.3 ± 0.1	4.52 ± 0.04	3.0 ± 0.1
doxorubicin	7.6 ± 0.3	0.71 ± 0.02	0.46 ± 0.02	5.43 ± 0.94

**Table 4:** Minimal inhibition concentration (MIC) values (μM) of monodentate ligands, [RhCp*(phen)Cl]Cl, and [RhCp*(8HQH_−1_)Cl] complexes and the mixed-ligand complexes in different bacterial strains after one day at pH 6 and 7.

	*S. aureus*		MRSA		*E. coli*		*E. coli* (Resistant)	
Compounds	pH 6	pH 7	pH 6	pH 7	pH 6	pH 7	pH 6	pH 7
econ	Bacteria were not able to proliferate at pH = 6 or below.	0.78	12.5	6.25	>100	>100	>100	>100
[RhCp*(phen)Cl]Cl		>100	>100	>100	>100	>100	>100	>100
+econ		0.78	12.5	3.125	25	50	50	>100
+mim		>100	>100	>100	>100	>100	>100	>100
+Py		>100	>100	>100	>100	>100	>100	>100
[RhCp*(8HQH_-1_)Cl]		12.5	12.5	12.5	>100	>100	>100	>100
+econ		0.39	6.25	3.125	25	25	>100	>100
+mim		12.5	25	12.5	>100	>100	>100	>100
+Py		12.5	50	12.5	>100	>100	>100	>100

## Data Availability

Not applicable.

## Supplementary Materials

The following supporting information can be downloaded at: https://www.mdpi.com/article/10.3390/pharmaceutics15020356/s1. Figure S1: Stability of the premixed complex under the same conditions as in biological studies; Figures S2–S14: ^1^H NMR and ^13^C NMR spectroscopic data of the isolated complexes; Figures S15–S20: Data quality for single crystal X-ray diffraction measurements; Figure S21: pH-dependent UV-vis spectra of [Ru(*p*-cym)(8HQH_-1_)(H_2_O)]^+^; Figure S22: pH-dependent UV-vis spectra of benzimidazole; Figure S23: Time-dependent UV-vis spectra of the *c*([RhCp*(en)(H_2_O)]^2+^):*c*(1-methylimidazole) = 1:2 mixture at pH = 5.94; Figure S24: Kinetics of Py liberation from synthesized complex; Figure S25: Fractions of the formed mixed ligand complexes of 1-methylimidazole in the pH-range 5.0–8.0; Figure S26: Fractions of [Ru(*p*-cym)(8HQH_-1_)(mim)]^+^ in the pH-range 5.0–8.0 at various concentrations of monodentate ligand and complex; Figure S27: Comparison of the M–N(monodentate) distances in different [M(arene)(N,N/O)(N)]-type complexes; Table S1: p*K*_a_ values of the co-ordinated water molecules in complexes; Table S2: Formation constants of mixed-ligand complexes; Tables S3–S5: Experimental data tables for single-crystal XRD; Tables S6 and S7: MIC values for all compounds, pH = 5–8.
