# Supplementary material for: Half-Sandwich Rhodium Complexes with Releasable N-Donor Monodentate Ligands: Solution Chemical Properties and the Possibility for Acidosis Activation
Source: Pharmaceutics. 2023 Jan 20;15(2):356. doi: 10.3390/pharmaceutics15020356 (PMC9964319; doi:10.3390/pharmaceutics15020356)
Supplement: Supplementary file 1 [file pharmaceutics-15-00356-s001.zip › pharmaceutics-2128187-supplementary.pdf]

# SUPPLEMENTARY MATERIAL

## Half-Sandwich Rhodium Complexes with Releasable N-Donor Monodentate Ligands: Solution Chemical Properties and Possibility for Acidosis Activation

János P. Mészáros [1,2,*], Wolfgang Kandioller [3,4], Gabriella Spengler [1,5],

Alexander Prado-Roller [3,4], Bernhard K. Keppler [3,4] and Éva A. Enyedy [1,2,*]

1 MTA-SZTE Lendület Functional Metal Complexes Research Group, University of Szeged, Dóm tér 7, H-6720 Szeged, Hungary
2 Department of Inorganic and Analytical Chemistry, Interdisciplinary Excellence Centre, University of Szeged, Dóm tér 7, H-6720 Szeged, Hungary
3 Institute of Inorganic Chemistry, Faculty of Chemistry, University of Vienna, Währinger Str. 42, A-1090 Vien-na, Austria
4 Research Cluster "Translational Cancer Therapy Research", University of Vienna, Währinger Str. 42, A-1090 Vienna, Austria
5 Department of Medical Microbiology, Albert Szent-Györgyi Health Center and Albert Szent-Györgyi Medical School, University of Szeged, Semmelweis utca 6, H-6725 Szeged, Hungary
* Correspondence: meszaros.janos@chem.u-szeged.hu (J.P.M.); enyedy@chem.u-szeged.hu (É.A.E.)

## CONTENTS

Figure S1. ........ 2

Characterization of the isolated complexes: $^{1}$H NMR and $^{13}$C NMR spectroscopic data ........ 3

data quality of structure determination, comparison of M–N bond lengths ........ 16

UV-vis spectra, fractions of mixed-ligand complexes as the function of pH, comparison of M–N bond lengths........ 19

Tables: Proton dissociation constants and formation constants ........ 26

Experimental data tables for single-crystal XRD ........ 27

MIC values for all compounds, pH = 5–8 ........ 30

References........ 31

## FIGURE S1.

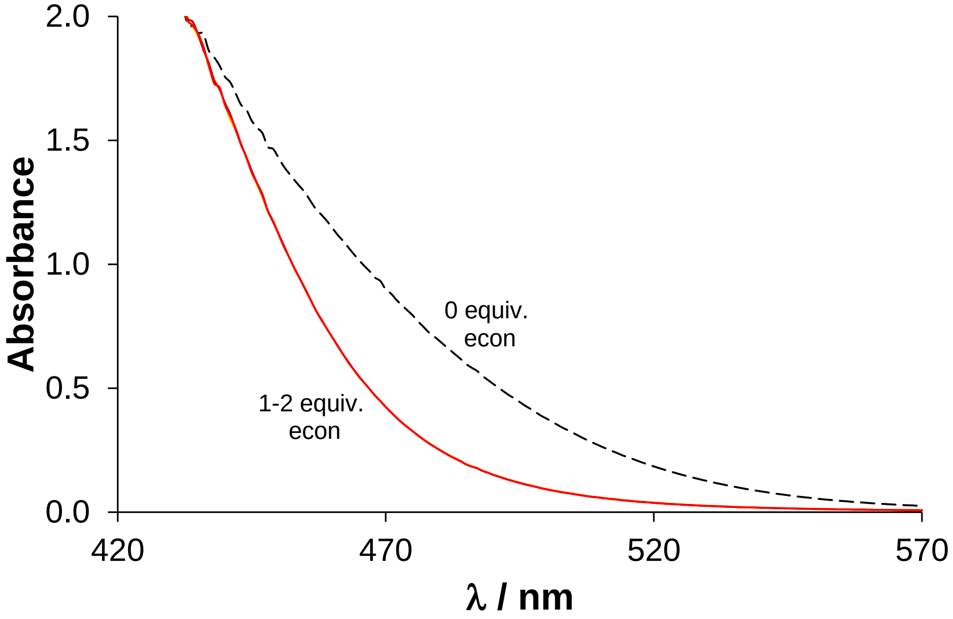


**Figure S1.** UV-vis spectra of the $[RhCp^*(8HQH_{-1})(H_2O)]^+$ complex at different concentrations of econazole. Conditions are the same as in the stock solutions prepared for anticancer and antibacterial experiments (pH = 7.4 (phosphate buffer) with 50% (v/v) ethanol). {$c([RhCp^*(8HQH_{-1})(H_2O)]^+)$ = 5 mM; *c*(econazole) = 0, 5 7.5 or 10 mM; $t$ = 25 °C; $\ell$ = 0.2 cm}

# CHARACTERIZATION OF THE ISOLATED COMPLEXES: $^{1}$H NMR AND $^{13}$C NMR SPECTROSCOPIC DATA

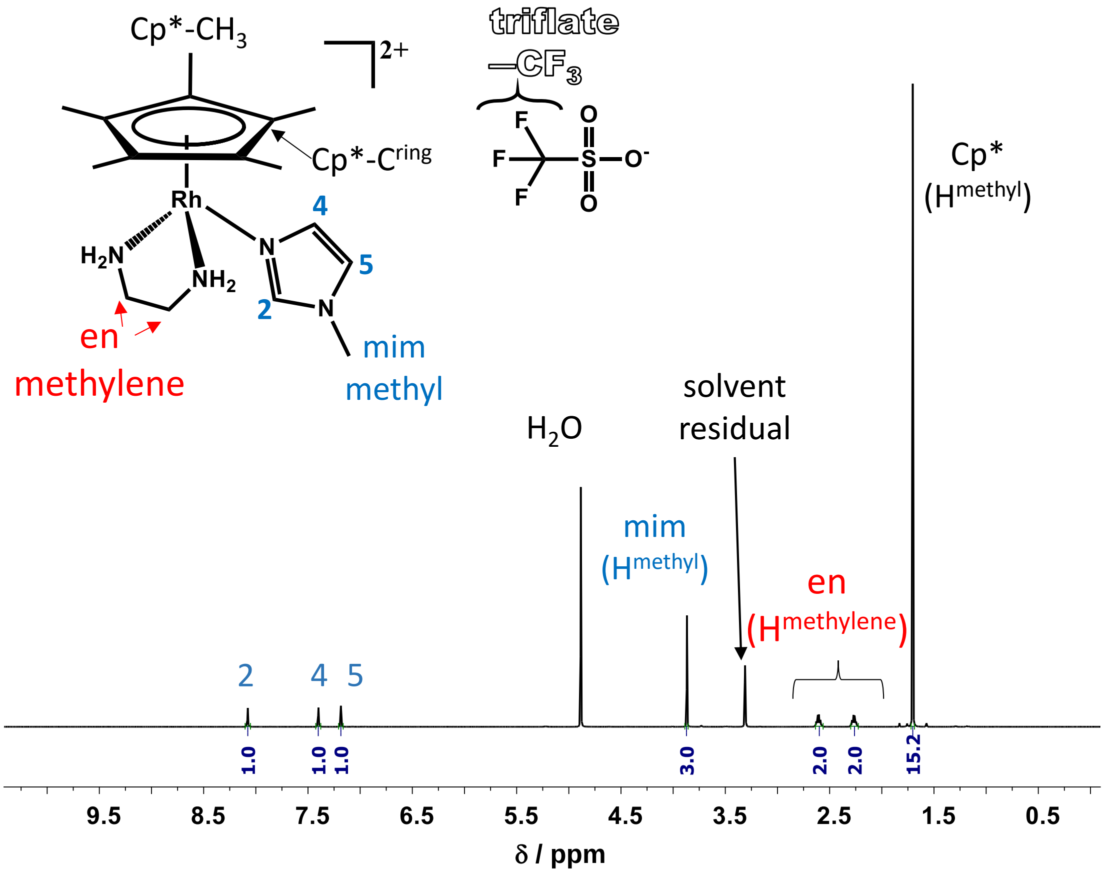


**Figure S2.** $^{1}$H NMR spectrum of [RhCp*(en)(mim)]($CF_3SO_3$)$_2$ in $CD_3OD$. Inserted structure shows numbering of peaks. {*c*(complex) = 10 mM, *t* = 25.0°C}

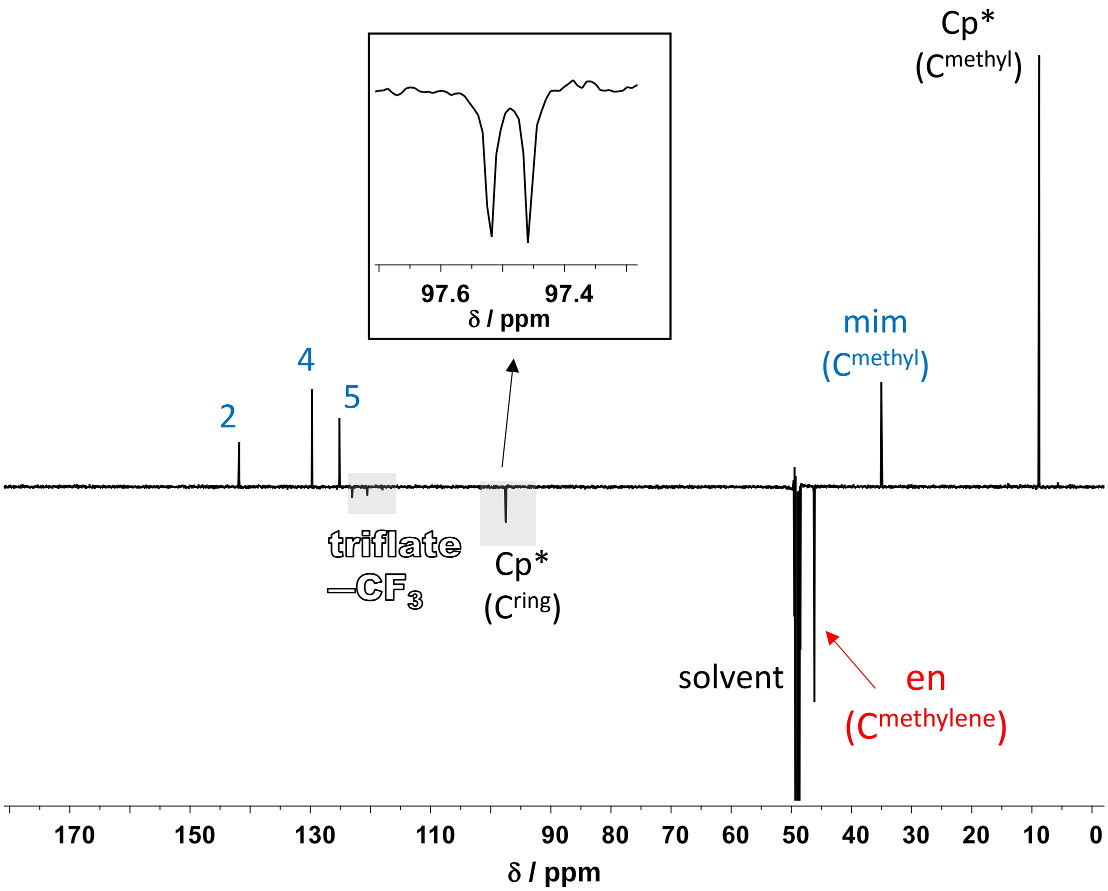


**Figure S3.** $^{13}C$ APT NMR spectrum of $[RhCp^*(en)(mim)](CF_3SO_3)_2$ in $CD_3OD$. For numbering see **Figure S2**. Inserted figure shows the doublet of **Cp*** ring carbon atoms as a result of coupling with $^{103}Rh$. {$c$(complex) = 10 mM, $t$ = 25.0°C}

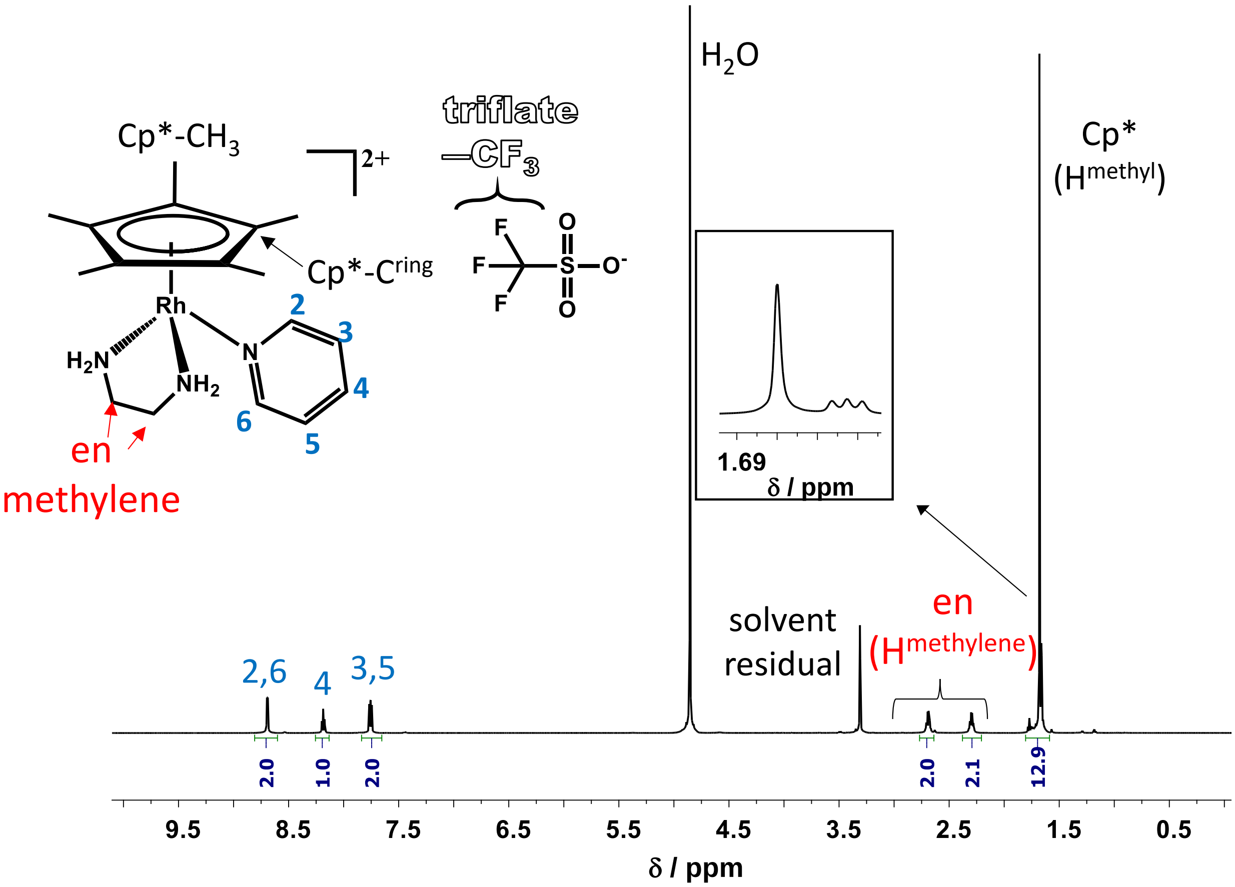


**Figure S4.** $^1H$ NMR spectrum of $[RhCp^*(en)(Py)](CF_3SO_3)_2$ in $CD_3OD$. Inserted structure shows numbering of peaks. Inset shows a triplet indicating the presence of $–CH_2D$ group. {$c$(complex) = 10 mM, $t$ = 25.0°C}

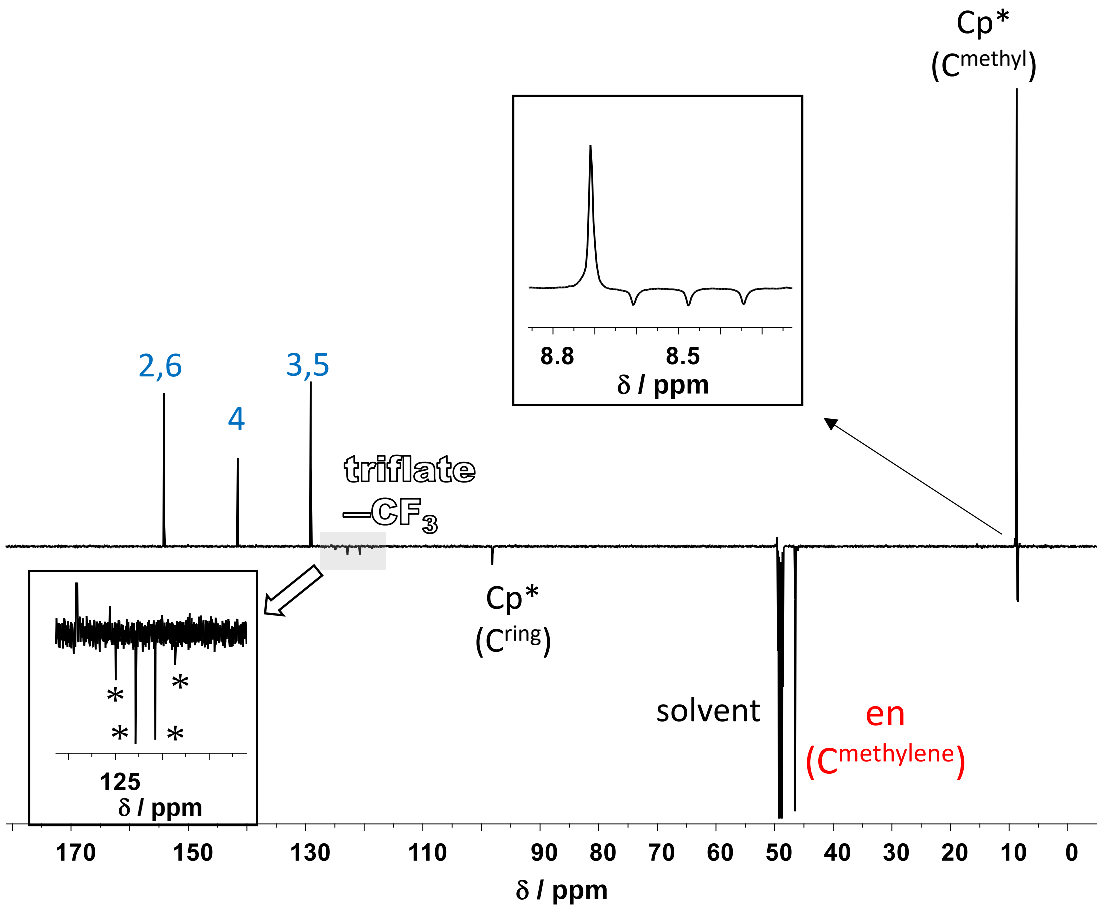


**Figure S5.** $^{13}C$ APT NMR spectrum of $[RhCp^*(en)(Py)](CF_3SO_3)_2$ in $CD_3OD$. For numbering see **Figure S4**. Inserted figure shows the quartet of **$-CF_3$** carbon atom as a result of coupling with $^{19}F$ and a triplet indicating the presence of $-CH_2D$ group. {*c*(complex) = 10 mM, *t* = 25.0°C}

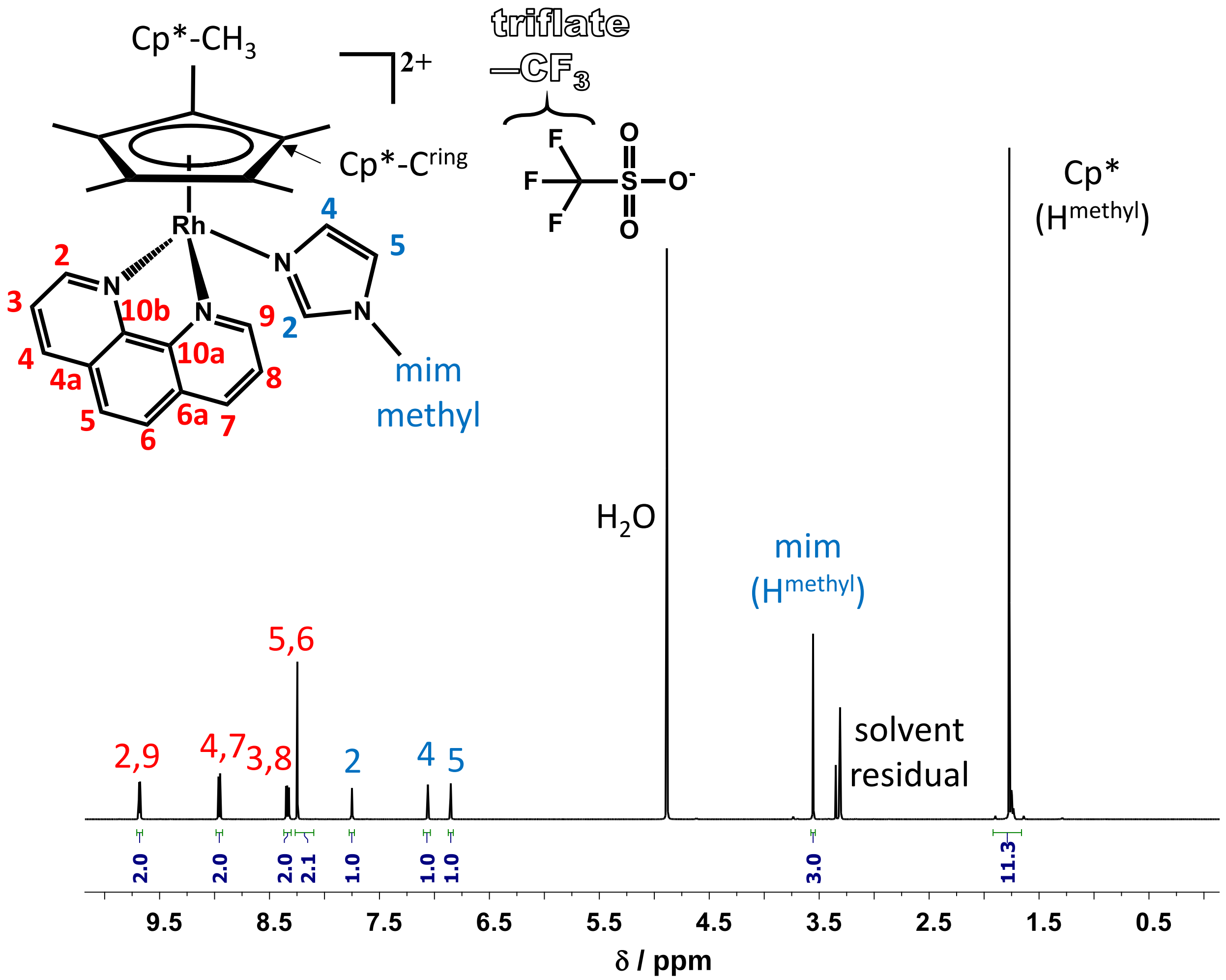


**Figure S6.** $^{1}H$ NMR spectrum of $[RhCp^*(phen)(mim)](CF_3SO_3)_2$ in $CD_3OD$. Inserted structure shows the numbering of peaks. {$c$(complex) = 10 mM, $t$ = 25.0°C}

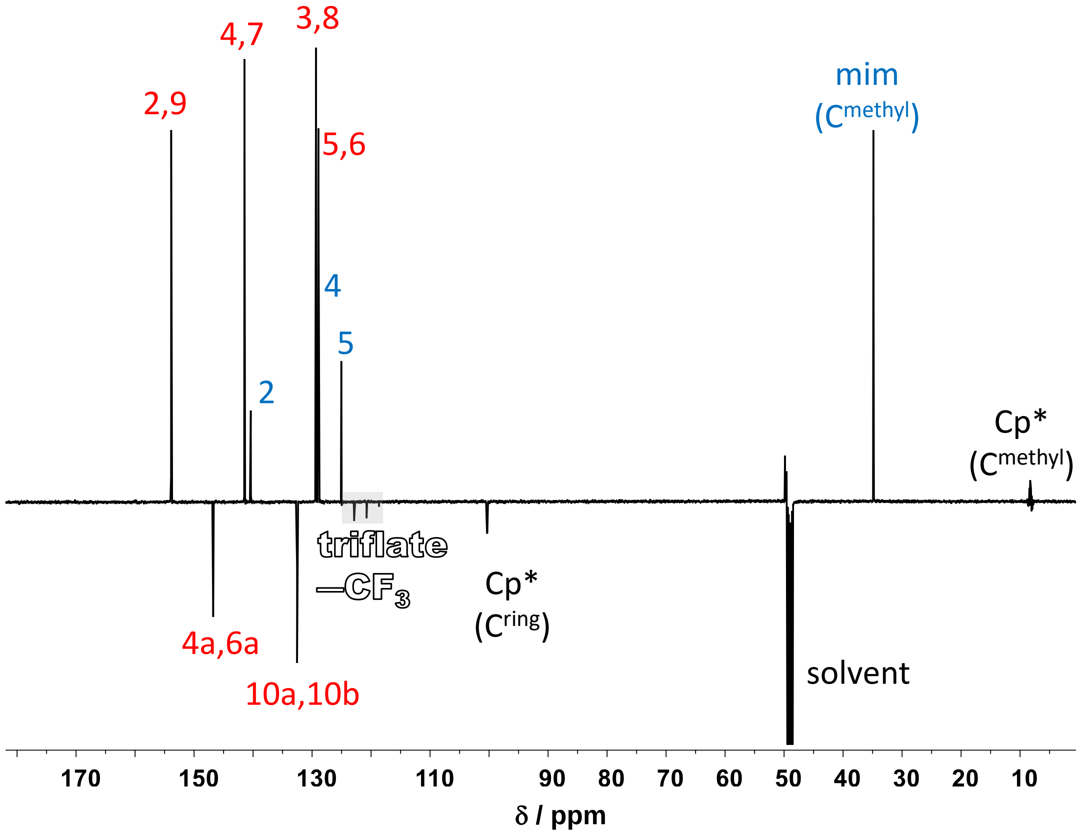


**Figure S7.** $^{13}C$ APT NMR spectrum of [RhCp*(phen)(mim)]$(CF_3SO_3)_2$ in $CD_3OD$. For numbering see **Figure S6**. {*c*(complex) = 10 mM, *t* = 25.0°C}

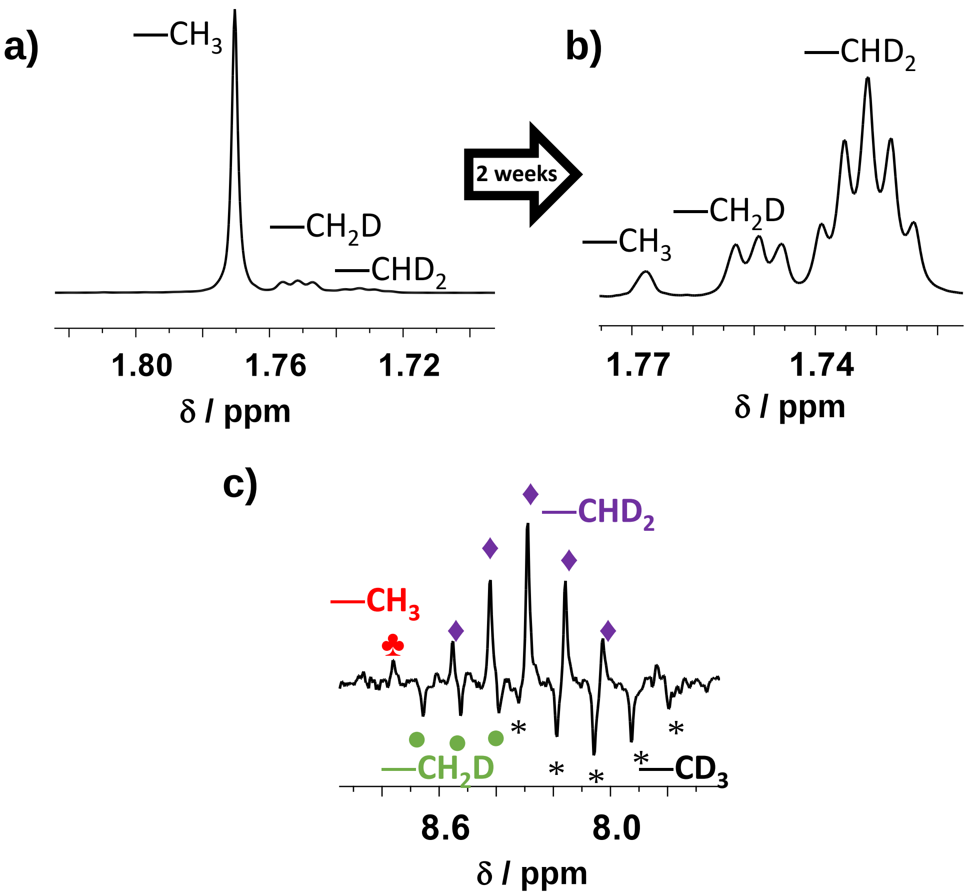


**Figure S8.** Upfield region of the $^1H$ NMR spectrum of $[RhCp^*(phen)(mim)](CF_3SO_3)_2$ in $CD_3OD$. a) Freshly prepared sample (waiting time < 1 day prior to measurement) and b) after ~2 weeks. c) Upfield region of the compound's $^{13}C$ NMR spectrum shows the presence of the **Cp*** methyl groups at different deuterated stages measured 2-week-old sample. {*c*(complex) = 10 mM, *t* = 25.0°C}

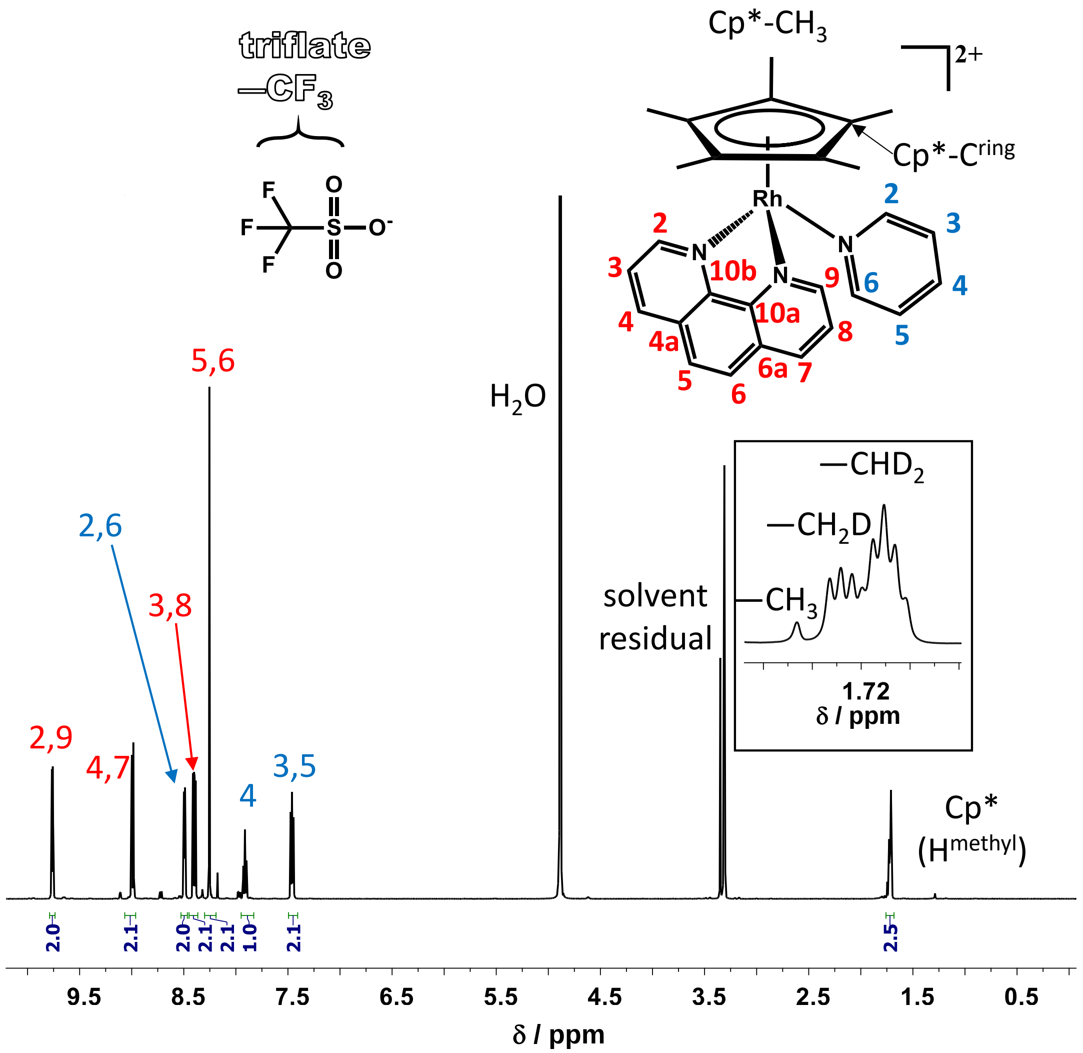


**Figure S9.** $^{1}$H NMR spectrum of $[RhCp^*(phen)(Py)](CF_3SO_3)_2$ in $CD_3OD$. Inserted structure shows numbering of peaks. Inset shows the methyl groups of **Cp*** at different deuteration stages. {*c*(complex) = 10 mM, *t* = 25.0°C}

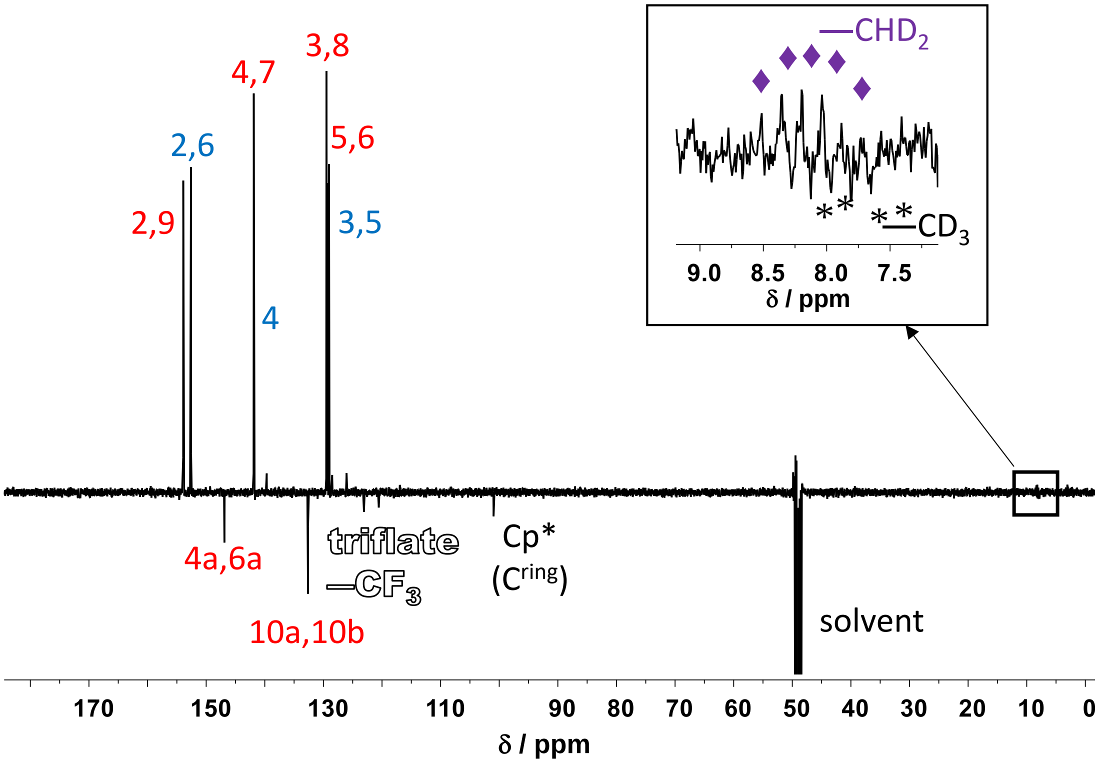


**Figure S10.** $^{13}C$ APT NMR spectrum of $[RhCp^*(phen)(Py)](CF_3SO_3)_2$ in $CD_3OD$. For numbering see **Figure S9**. Inset shows the presence of the **Cp*** methyl groups at different deuterated stages. {$c$(complex) = 10 mM, $t$ = 25.0°C}

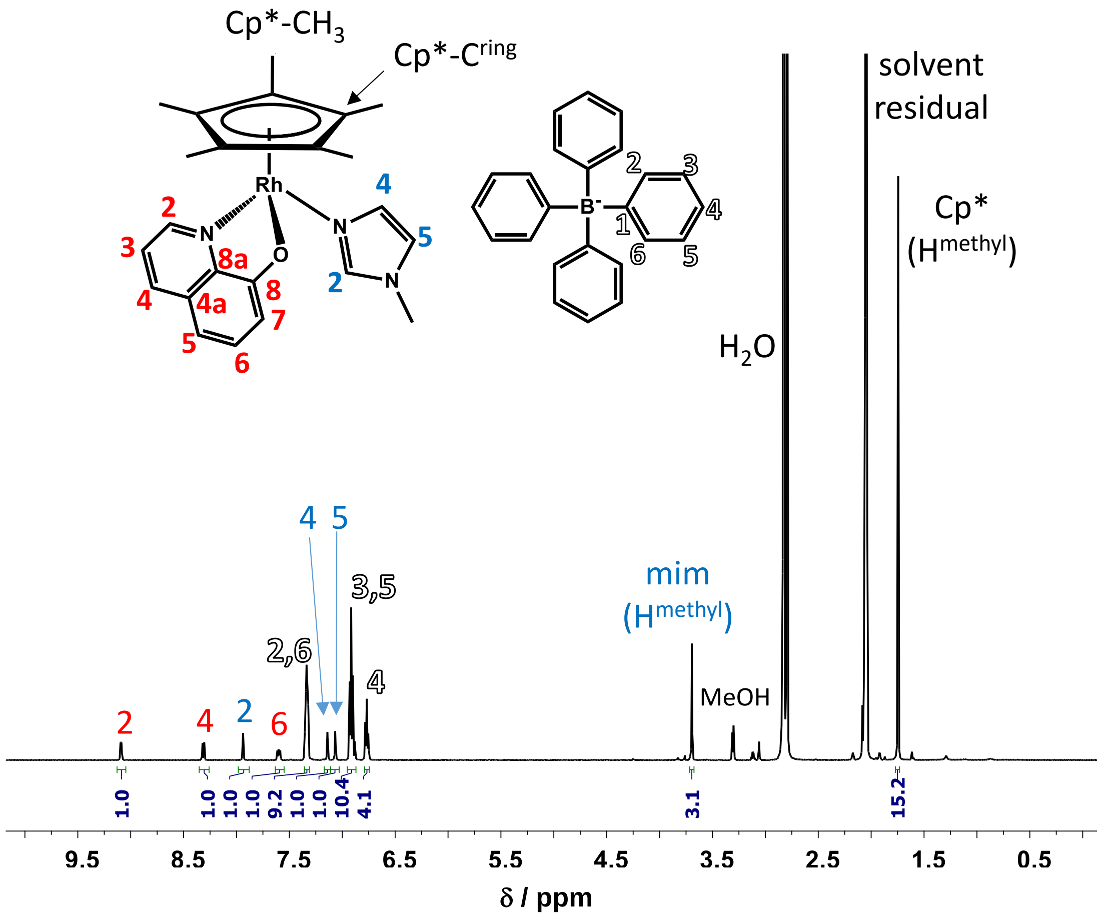


**Figure S11.** $^1$H NMR spectrum of [RhCp*(8HQH$_{-1}$)(mim)]($BPh_4$) in acetone-d$_6$. Inserted structure shows numbering of peaks. {*c*(complex) = 2 mM, *t* = 25.0°C}

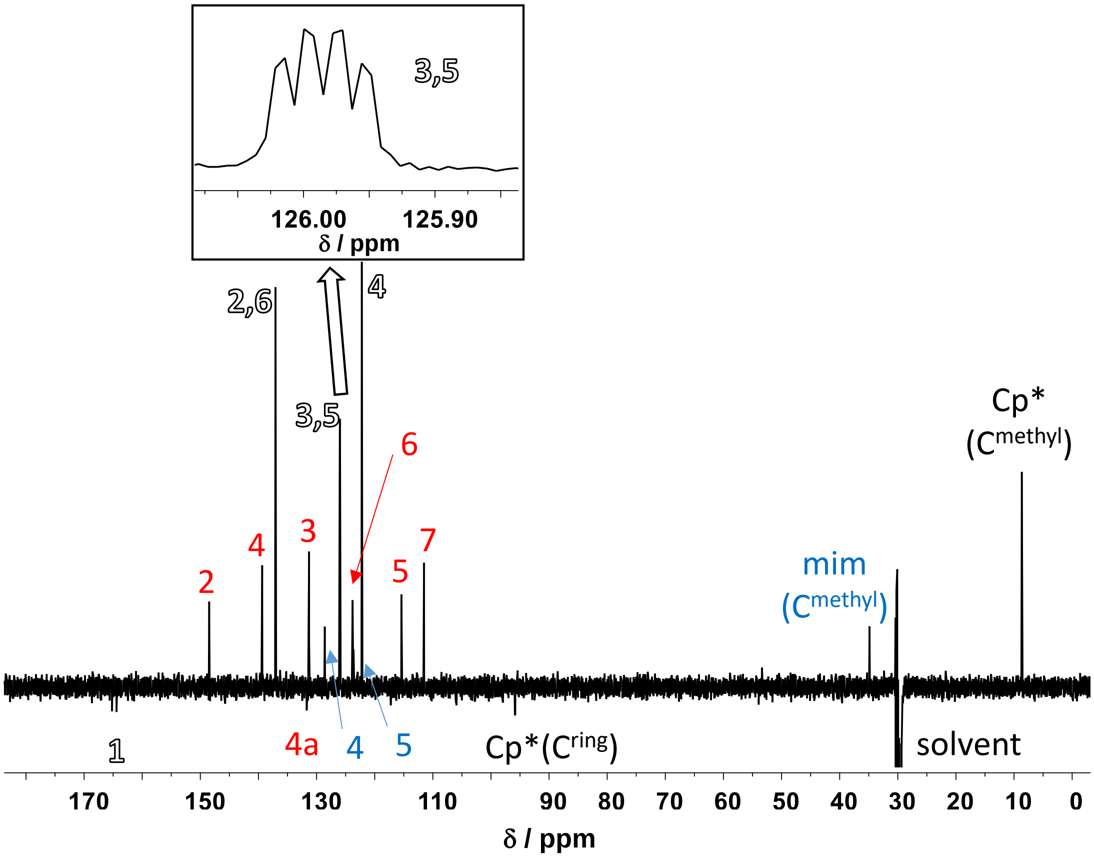


**Figure S12.** $^{13}C$ APT NMR spectrum of [RhCp*(8HQH$_{-1}$)(mim)](BPh$_4$) in acetone-d$_6$. For numbering see **Figure S11**. Inset shows the coupling with $^{11}B$ in $BPh_4^-$. {*c*(complex) = 2 mM, *t* = 25.0°C}

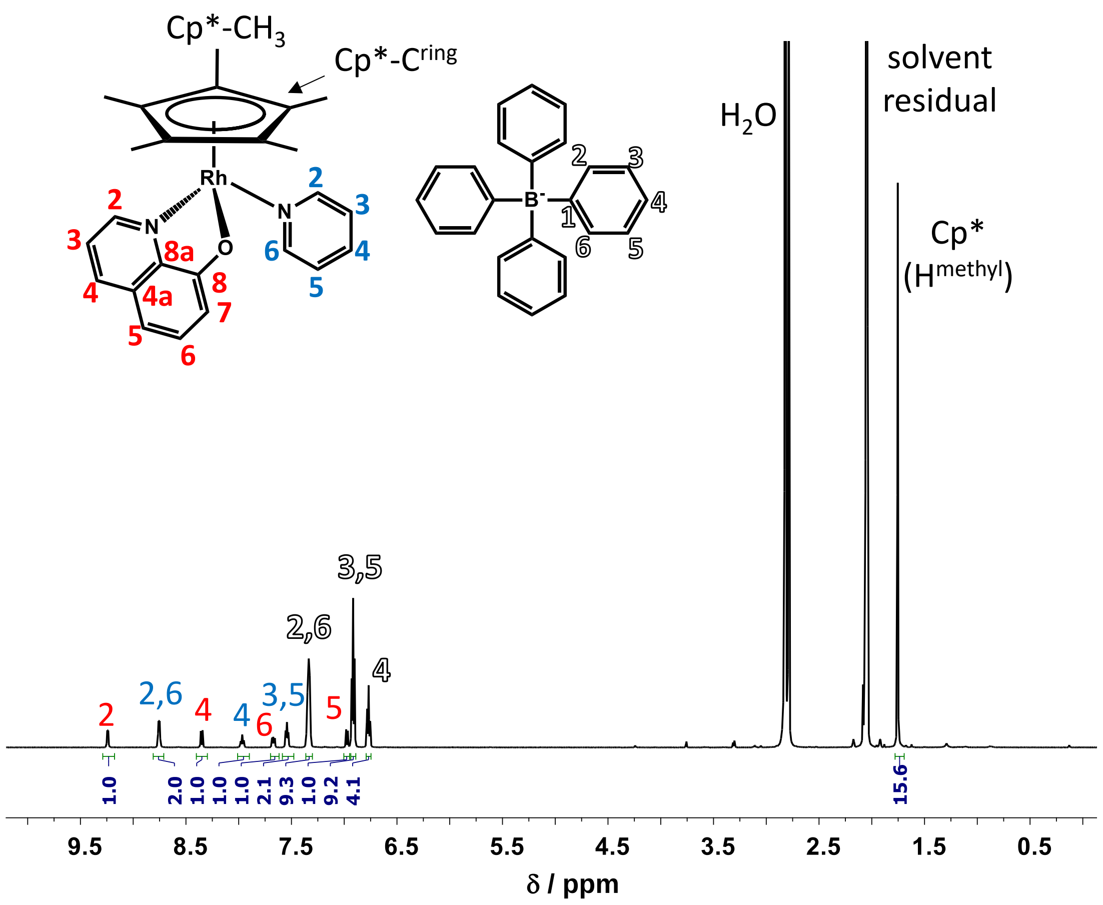


**Figure S13.** $^{1}H$ NMR spectrum of [RhCp*(8HQH$_{-1}$)(Py)](BPh$_4$) in acetone-d$_6$. Inserted structure shows numbering of peaks. {$c$(complex) = 2 mM, $t$ = 25.0°C}

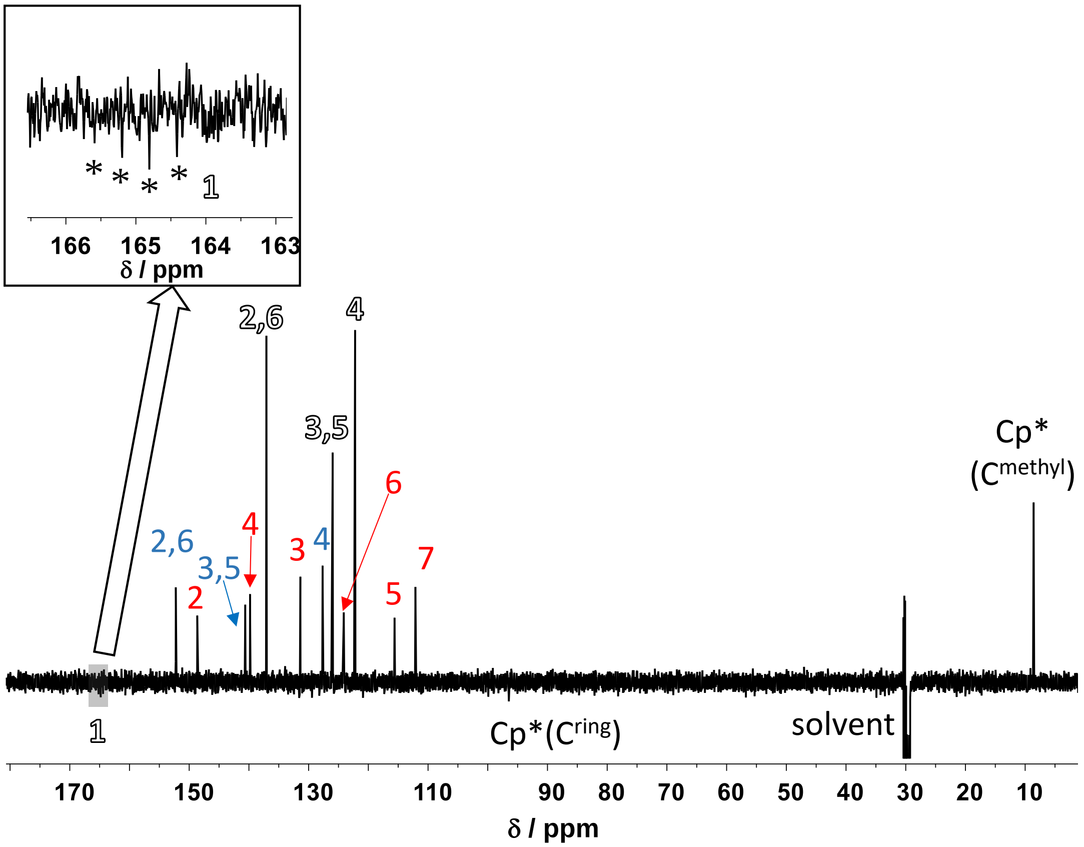


**Figure S14.** $^{13}C$ APT NMR spectrum of [RhCp*(8HQH$_{-1}$)(Py)]($BPh_4$) in acetone-d$_6$. For numbering see **Figure S13**. Inset shows the coupling with $^{11}B$ in $BPh_4^-$. {$c$(complex) = 2 mM, $t$ = 25.0°C}

## DATA QUALITY OF STRUCTURE DETERMINATION, COMPARISON OF M–N BOND LENGTHS

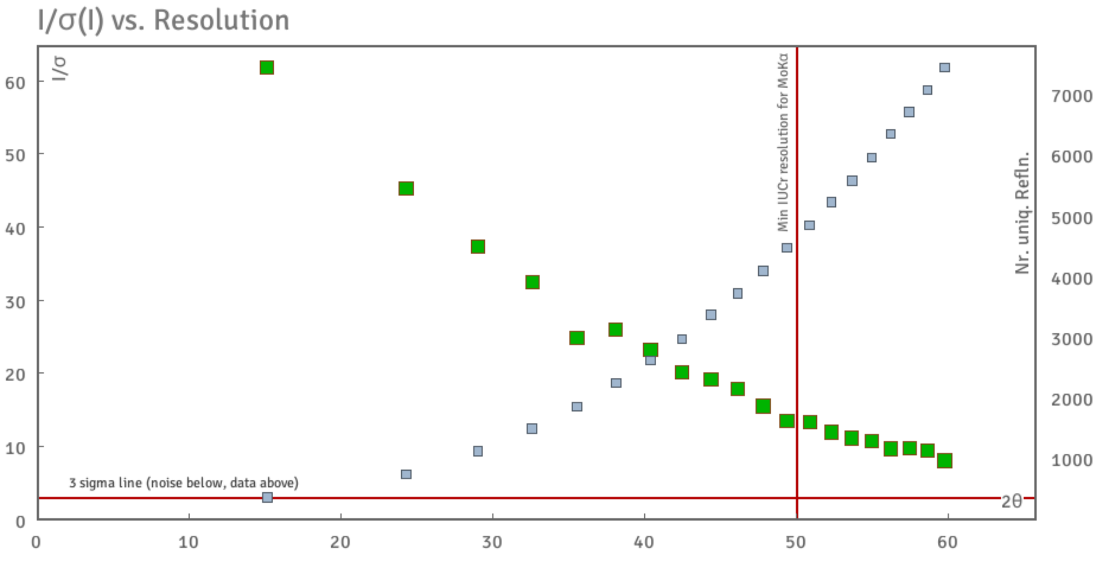


**Figure S15.** Data quality for complex **1′**: all data are above the three sigma line (along the minimum defined by the International Union of Crystallography (IUCR)).

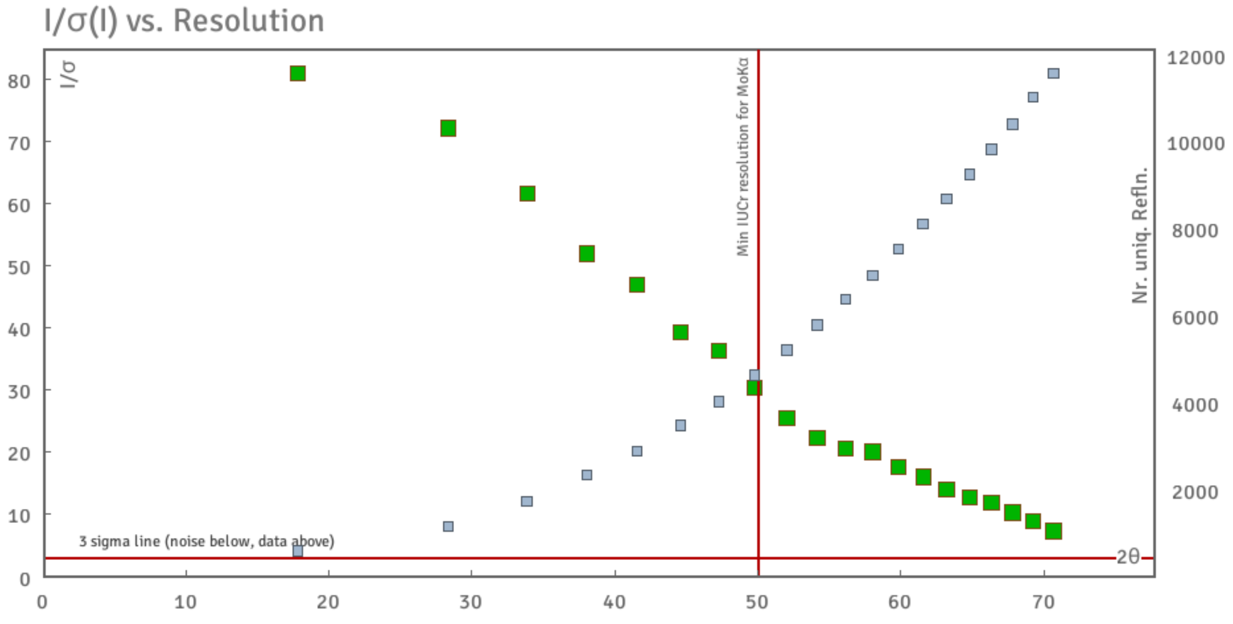


**Figure S16.** Data quality for complex **2′**: all data are above the three sigma line (along the minimum defined by IUCR).

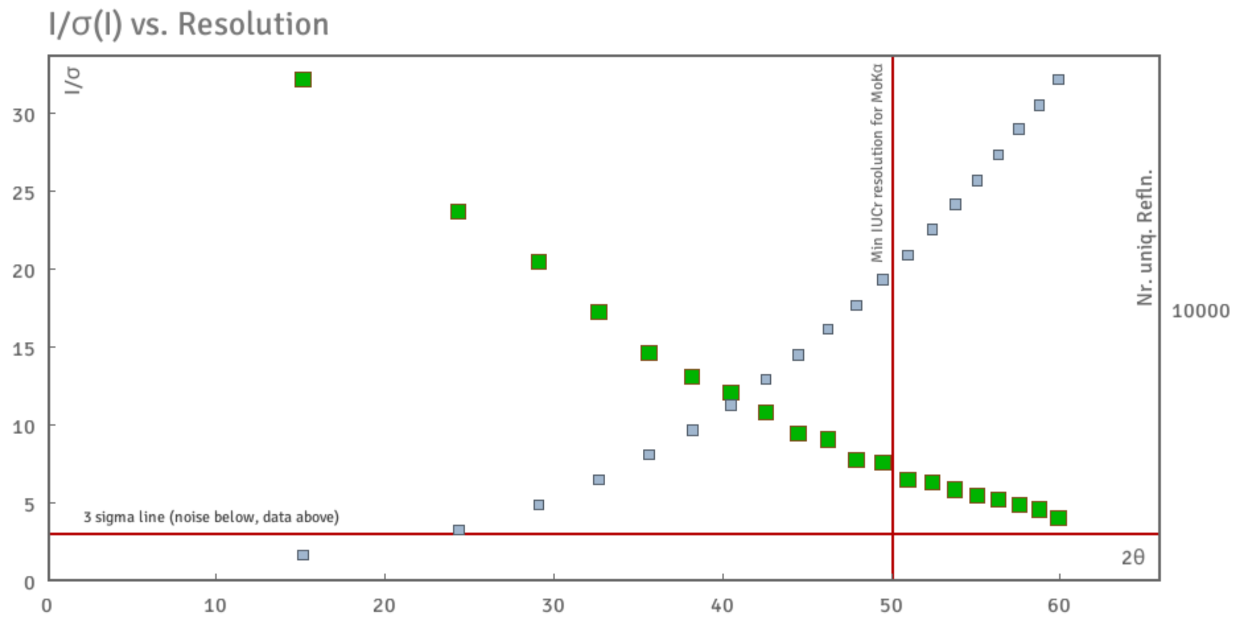


**Figure S17.** Data quality for complex **3′**: all data are above the three sigma line (along the minimum defined by IUCR).

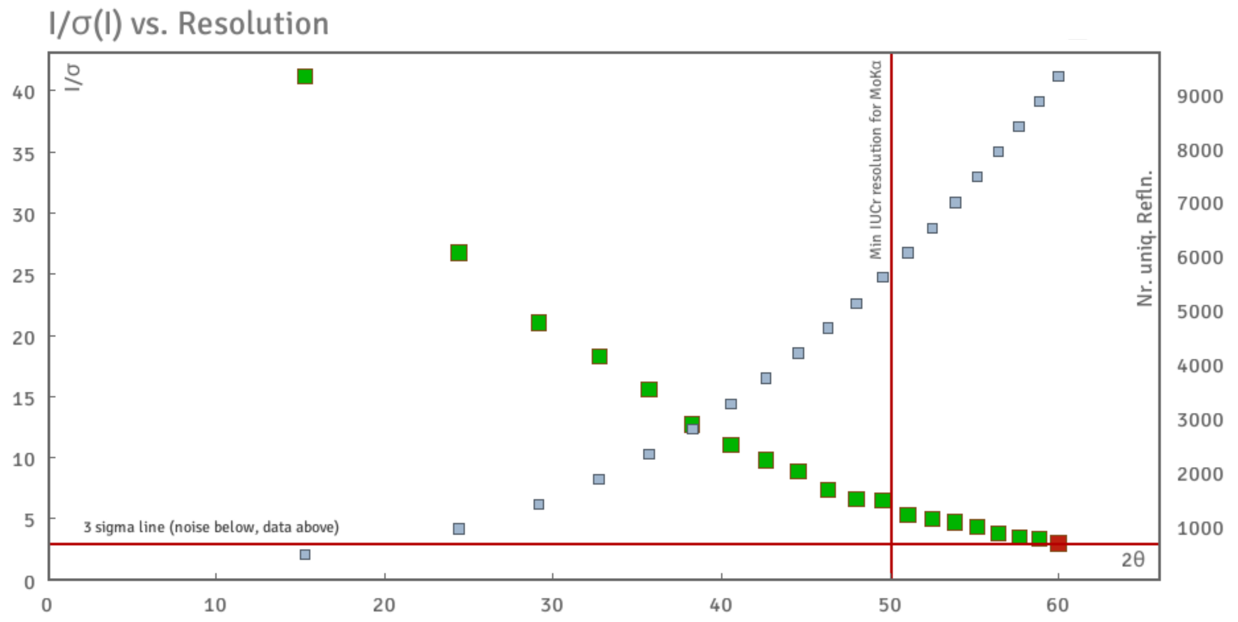


**Figure S18.** Data quality for complex **4′**: all data are above the three sigma line (along the minimum defined by IUCR).

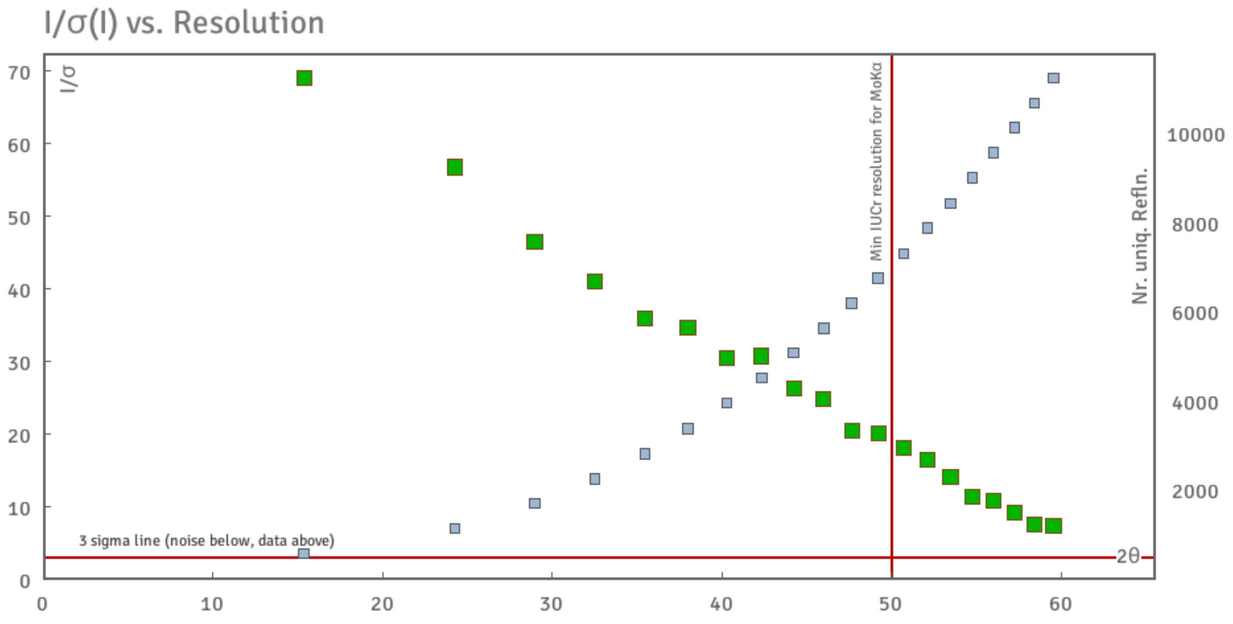


**Figure S19.** Data quality for complex **5′**: all data are above the three sigma line (along the minimum defined by IUCR).

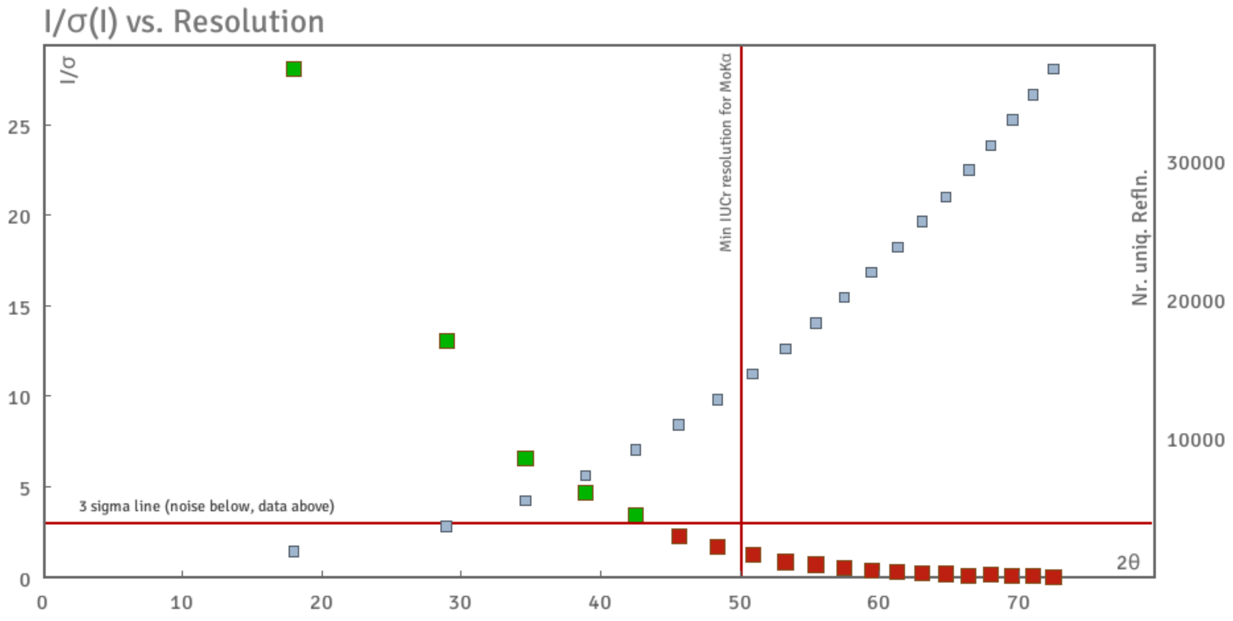


**Figure S20.** Data quality for complex **6′**: almost all data are above the three sigma line (along the minimum defined by IUCR). Data close to 50 degree (0.82, Mo) are still used in the refinement.

## UV-Vis SPECTRA, FRACTIONS OF MIXED-LIGAND COMPLEXES AS THE FUNCTION OF pH, COMPARISON OF M–N BOND LENGTHS

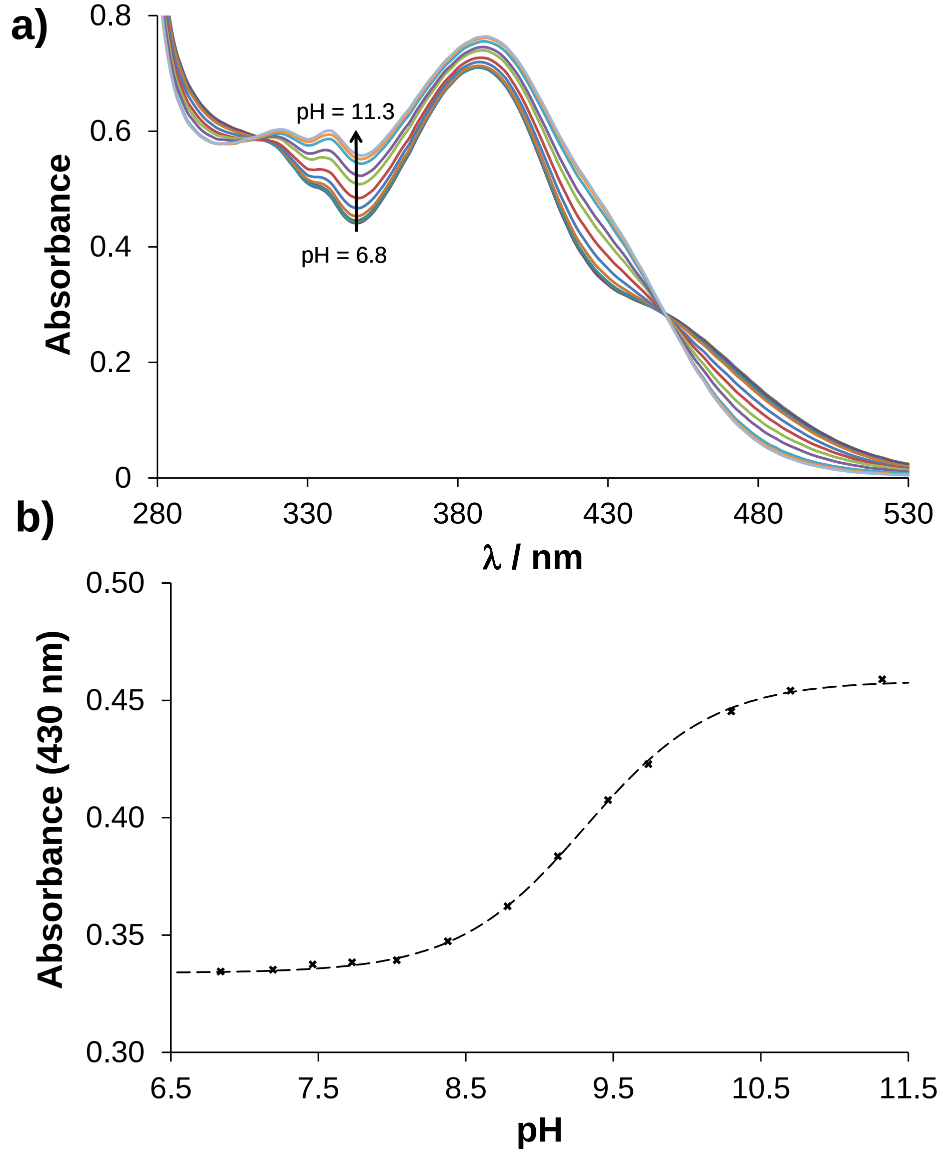


**Figure S21.** a) pH-dependent UV-vis spectra of $[Ru(\eta^6\text{-}p\text{-cym})(8HQH_{-1})(H_2O)]^+$ in the pH-range 2.0–11.5. b) Absorbance values at 430 nm in the function of pH. {$c([Ru(\eta^6\text{-}p\text{-cym})(8HQH_{-1})(H_2O)]^+)$ = 200 μM; $I$ = 0.10 M (KCl), $t$ = 25 °C, $\ell$ = 1 cm}

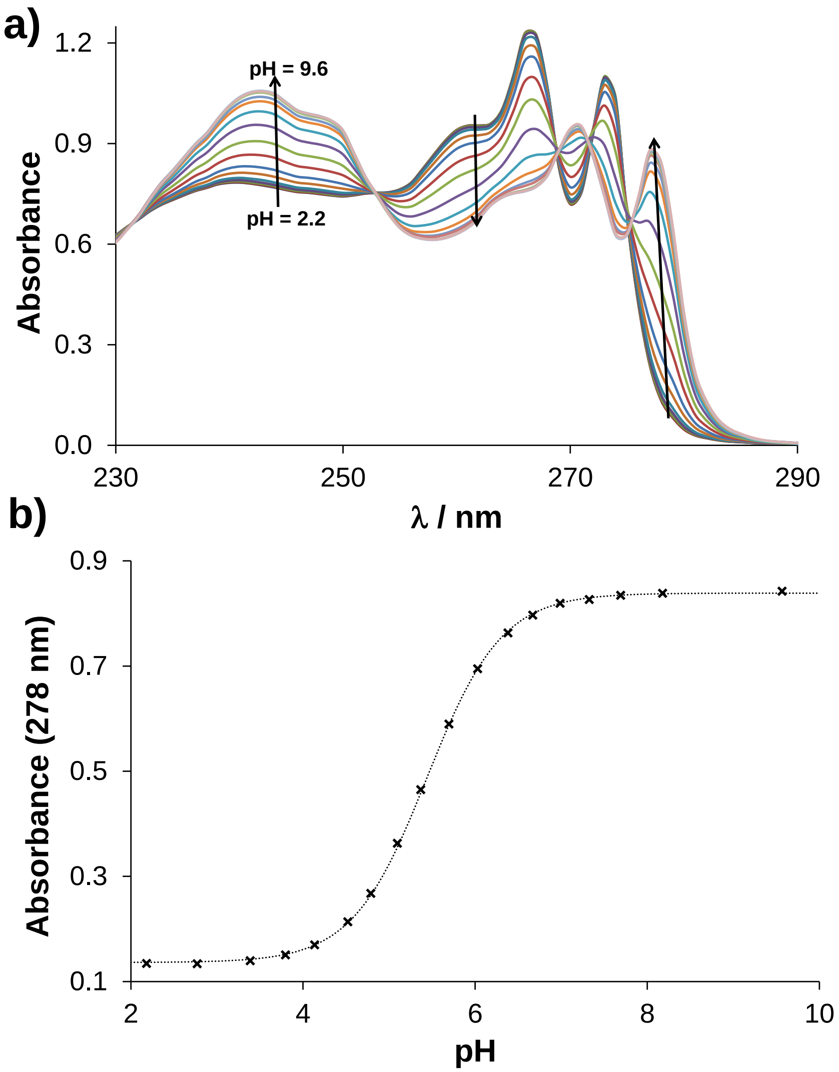


**Figure S22.** a) pH-dependent UV-vis spectra of benzimidazole in the pH-range 2.0–10.5. b) Absorbance values at 278 nm in the function of pH together with the fitted line (dotted line). {$c$(bim) = 200 μM; $I$ = 0.10 M (KCl), $t$ = 25 °C, $\ell$ = 0.2 cm}.

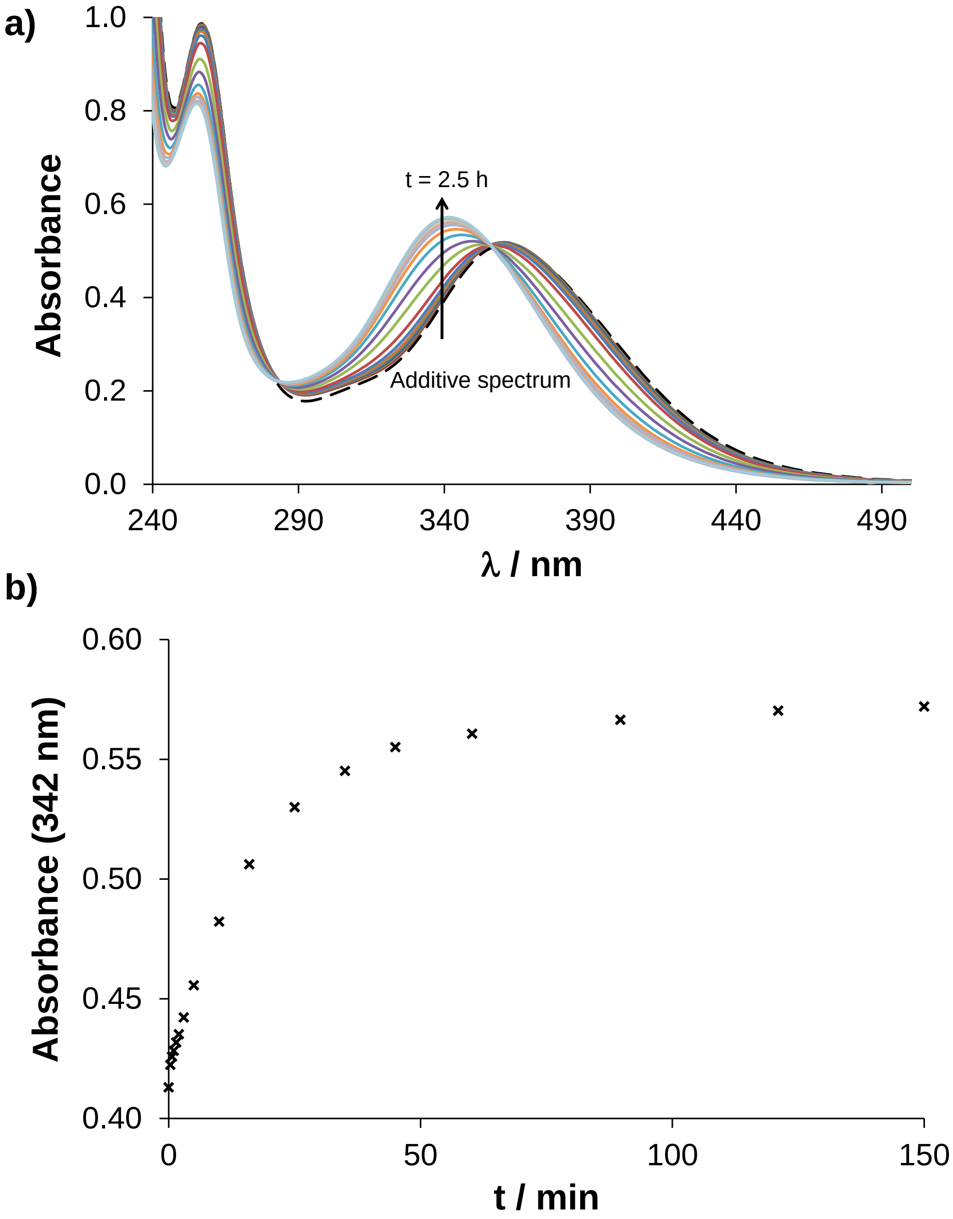


**Figure S23.** a) Time-dependent UV-vis spectra of the sample containing a mixture with $c([RhCp^{*}(en)(H_2O)]^{2+})$:$c$(1-methylimidazole) = 1:2 composition at pH = 5.94. b) Time dependence of absorbance values at 342 nm. {$c([RhCp^{*}(en)(H_2O)]^{2+})$ = 200 μM; $c$(1-methylimidazole) = 400 μM; pH = 5.94 (PBS′); $t$ = 25 °C; $\ell$ = 1 cm}

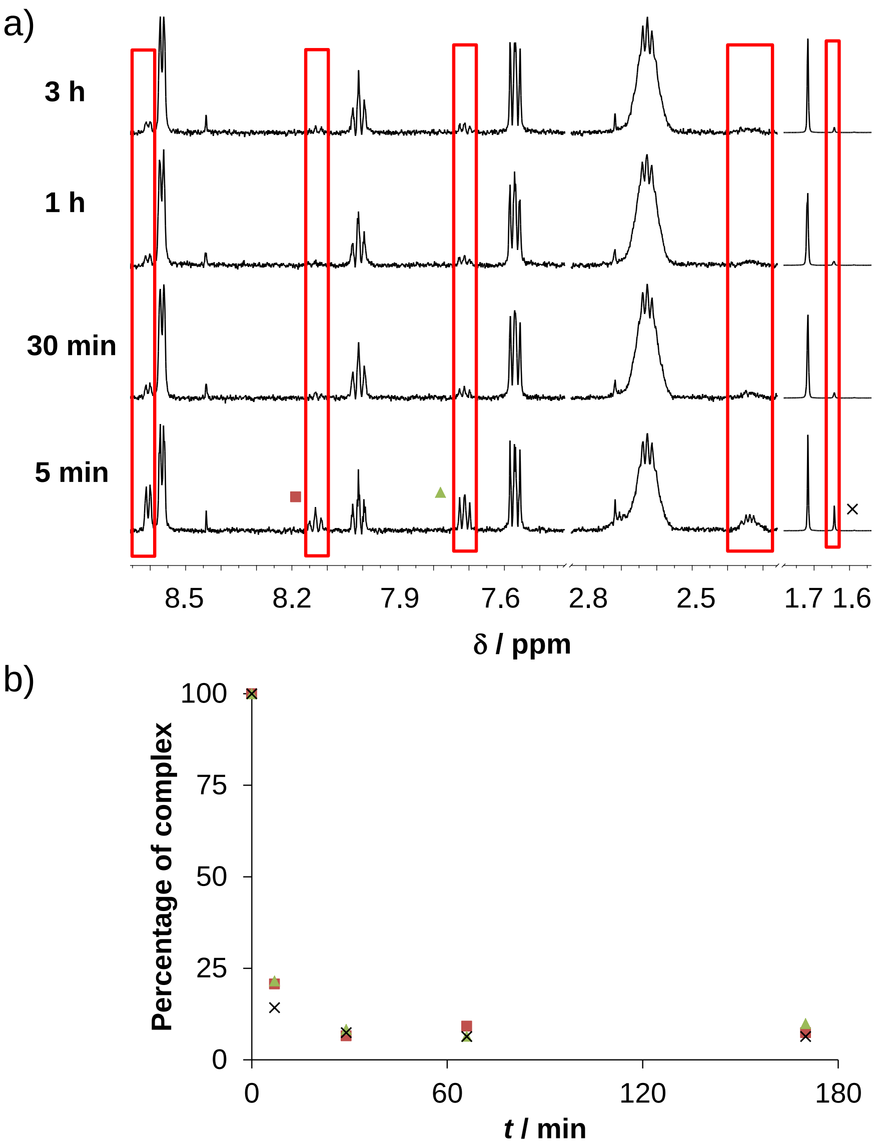


**Figure S24.** a) Time-dependent $^1H$ NMR spectra of the $[RhCp^*(en)(Py)](CF_3SO_3)_2$ synthesized complex at pH = 6.0. Red rectangles are highlighting peaks of the remaining mixed-ligand complex. b) Time-dependence of the presence of mixed-ligand complex (in percentage) based on integrals of the peaks of pyridine and Cp*. Assignation: × = Cp*; ■ = pyr-$\mathbf{H}^{3,5}$; ▲ = pyr-$\mathbf{H}^4$. {$c([RhCp^*(en)(Py)](CF_3SO_3)_2)$ = 200 μM; pH = 6.0 (PBS'); 10% $D_2O$; $t$ = 25 °C; }

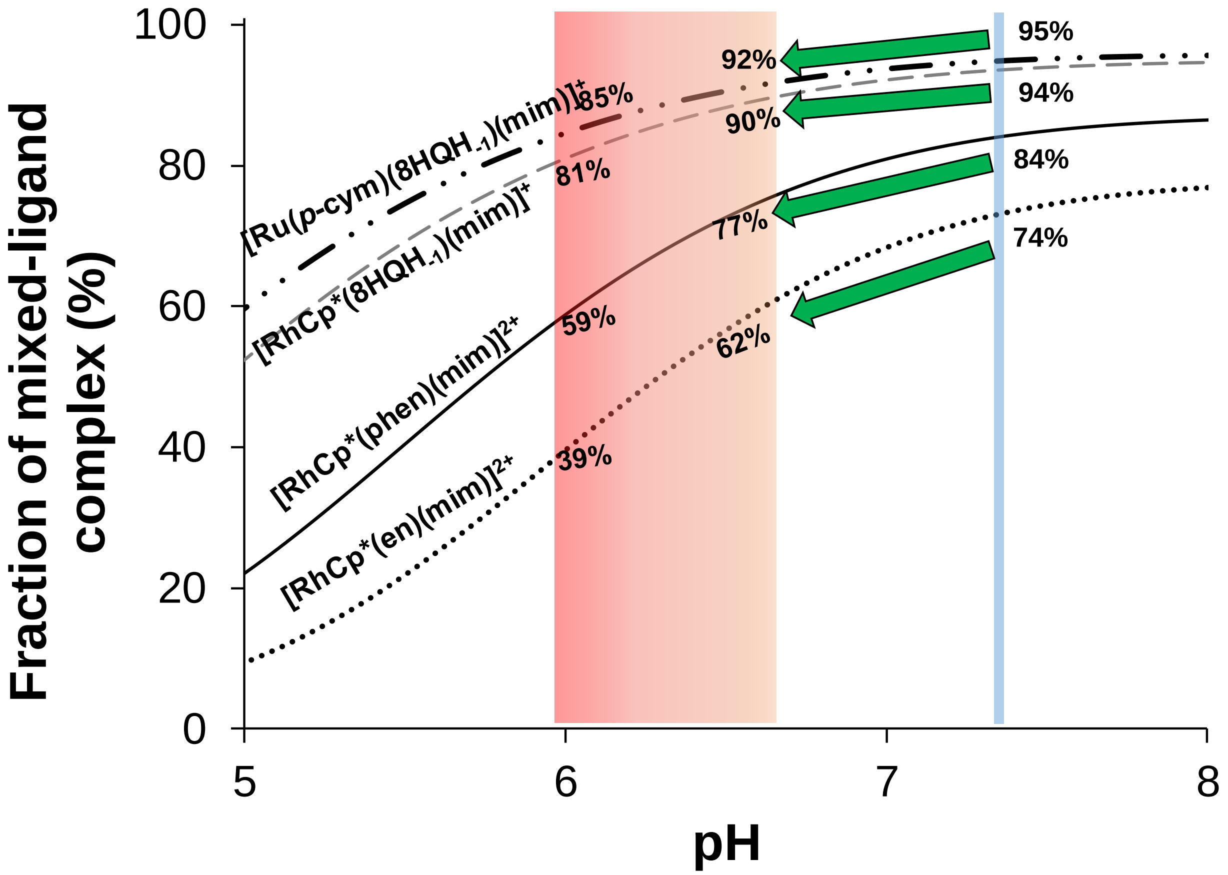


**Figure S25.** Fractions of the formed mixed-ligand complexes of 1-methylimidazole in the pH-range 5.0–8.0. Calculations based on the stability constants of the mixed-ligand complexes (**Table S2**) and proton dissociation constants in **Table 1**. {*c*([M(arene)(N,N/O)($H_2O$)) = *c*(mim) = 100 μM; *I* = 0.10 M (KCl), *t* = 25 °C}.

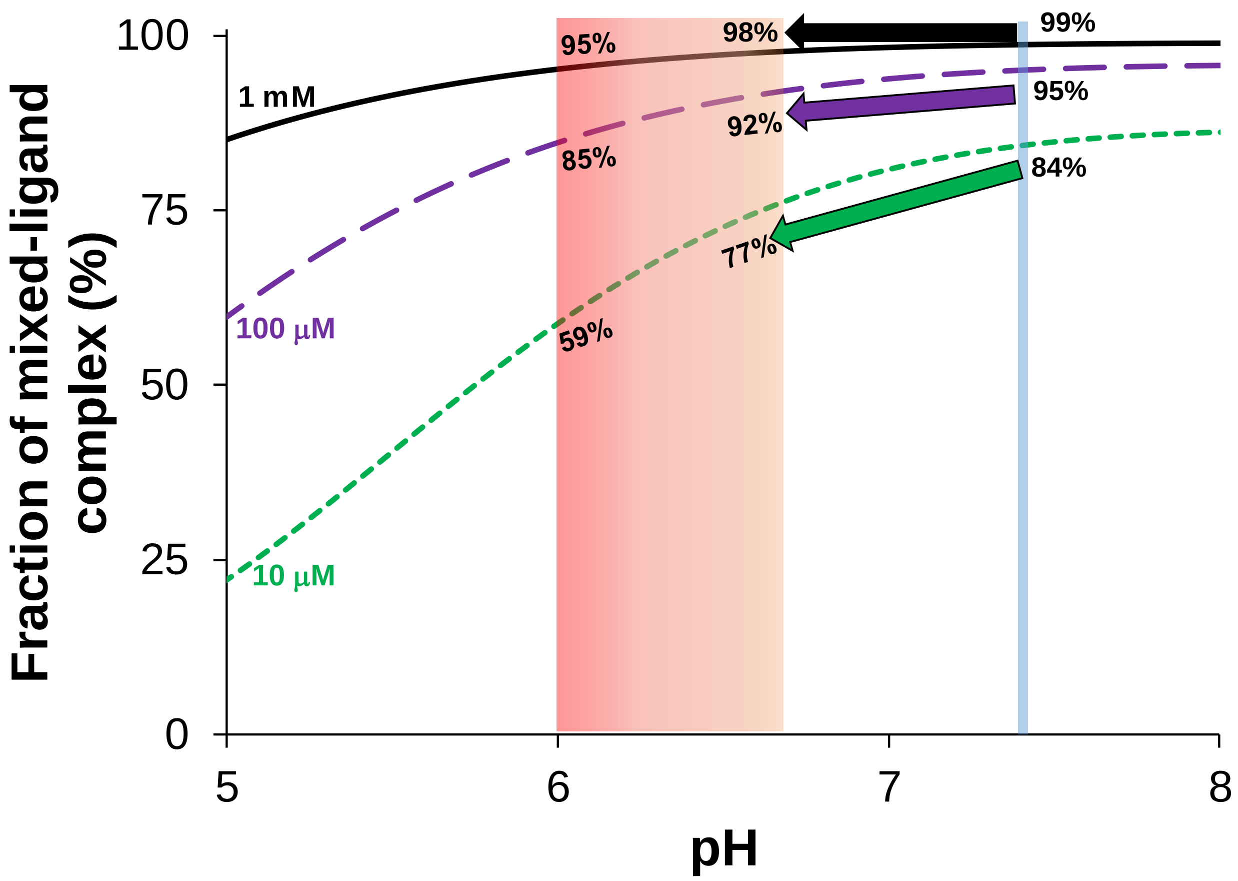


**Figure S26.** Formation (%) of [Ru($\eta^6$-*p*-cym)(8HQH$_{-1}$)(mim)]$^+$ in the pH-range 5.0–8.0 at various equimolar concentrations of monodentate ligand and aqua complex. {*c*([Ru($\eta^6$-*p*-cym)(8HQH$_{-1}$)($H_2O$)]$^+$) = *c*(mim) = 10 μM, 100 μM or 1 mM; *I* = 0.10 M (KCl), *t* = 25 °C}.

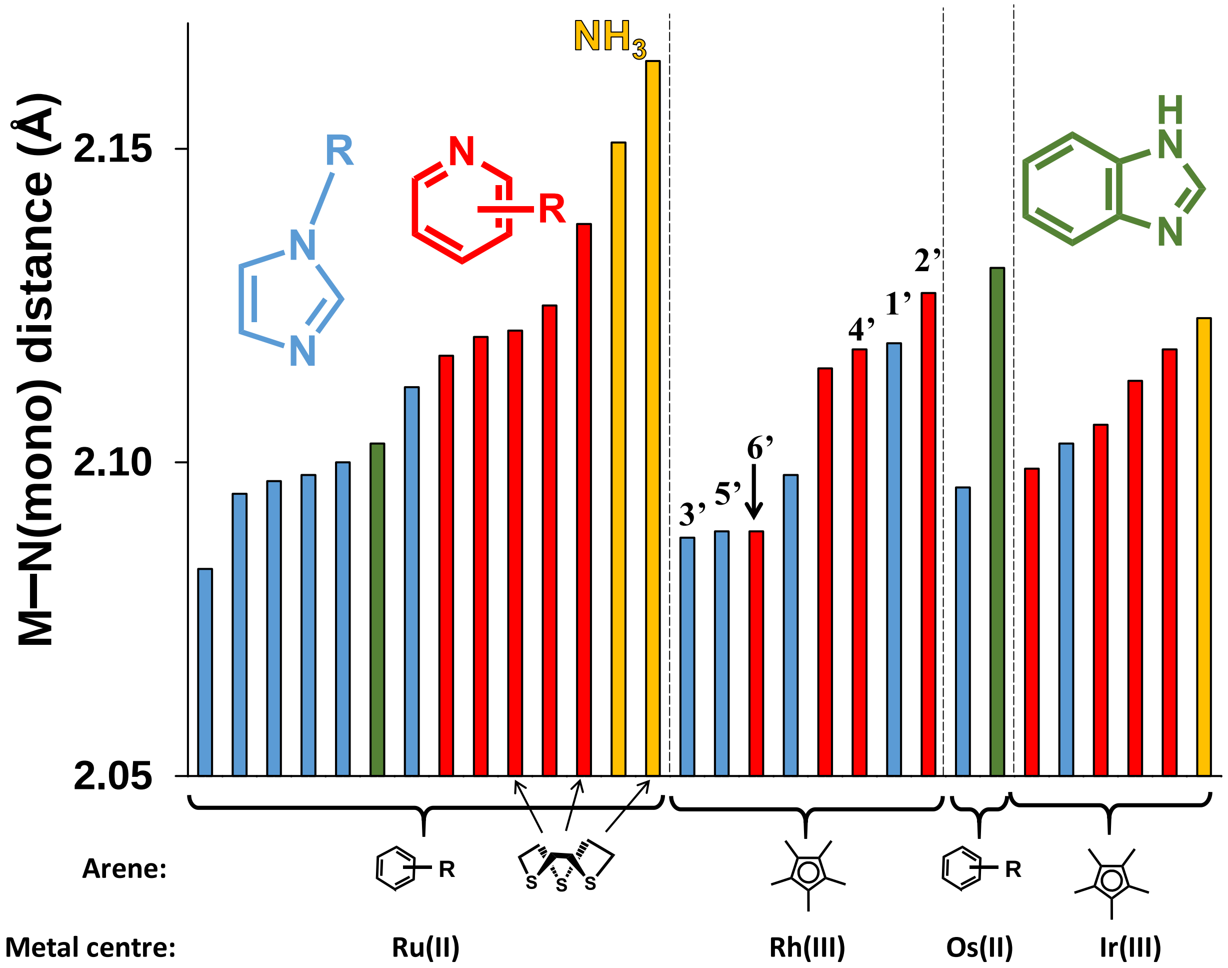


**Figure S27.** Comparison of the M–N(monodentate) distances in different [M(arene)(N,N/O)(N)]-type complexes reported in references [1-12]. Colour code for the monodentate ligand: blue: imidazole derivatives; red: pyridine derivatives; green: benzimidazole; yellow: $NH_3$. Roman numbers indicate the values derived from complexes **1'–6'**.

# TABLES: PROTON DISSOCIATION CONSTANTS AND FORMATION CONSTANTS

**Table S1.** Proton dissociation constants of the coordinated water molecules in $[M(arene)(N,N/O)(H_2O)]^{2+/+}$ complexes {$I$ = 0.10 M (KCl); $t$ = 25.0 °C}.

| Compound | p$K_a$ | Method | Literature data |
|---|---|---|---|
| $[RhCp^*(en)(H_2O)]^{2+}$ | 10.6±0.1 | UV-vis | 9.58[1] [13]<br>11.05[2] [13] |
| $[RhCp^*(8HQH_{-1})(H_2O)]^{+}$ | 10.6±0.1 | UV-vis | 10.27[2] [14] |
| $[RhCp^*(phen)(H_2O)]^{2+}$ | 10.05±0.01 | UV-vis | 8.58[2] [15] |
| [Ru(*p*-cym)(en)($H_2O$)]$^{2+}$ | 9.03±0.01 | UV-vis | 8.14[2] [15] |
| [Ru(*p*-cym)($8HQH_{-1}$)($H_2O$)]$^{+}$ | 9.31±0.01 | UV-vis | 9.19[2] [14] |
| [Ru(*p*-cym)(phen)($H_2O$)]$^{2+}$ | 8.91±0.01 | UV-vis | 7.59[2] [15] |

[1] $I$ = 0.20 M ($KNO_3$); [2] $I$ = 0.20 M (KCl)

**Table S2.** Formation constants (log$K$) determined for mixed-ligand half-sandwich complexes of Rh(III) and Ru(II) by UV-vis and $^1$H NMR spectroscopy {$I$ = 0.10 M (KCl); $t$ = 25.0 °C}.

**$[M(arene)(N,N/O)(H_2O)]^{2+/+} + N_{mono} \rightleftharpoons [M(arene)(N,N/O)(N_{mono})]^{2+/+} + H_2O$**

| Aqua complex | Monodentate ligand | log$K$ |
|---|---|---|
| $[RhCp^*(en)(H_2O)]^{2+}$ | bim | 3.3 ± 0.2 |
| $[RhCp^*(en)(H_2O)]^{2+}$ | mim | 4.2 ± 0.1 |
| $[RhCp^*(en)(H_2O)]^{2+}$ | Py | 2.6 ± 0.1 |
| $[RhCp^*(phen)(H_2O)]^{2+}$ | bim | 4.9 ± 0.2 |
| $[RhCp^*(phen)(H_2O)]^{2+}$ | mim | 5.5 ± 0.1 |
| $[RhCp^*(phen)(H_2O)]^{2+}$ | Py | 4.1 ± 0.1 |
| $[RhCp^*(8HQH_{-1})(H_2O)]^{+}$ | bim | 3.4 ± 0.1 |
| $[RhCp^*(8HQH_{-1})(H_2O)]^{+}$ | mim | 4.7 ± 0.2 |
| $[RhCp^*(8HQH_{-1})(H_2O)]^{+}$ | Py | 3.0 ± 0.1 |
| $[RuCym(en)(H_2O)]^{2+}$ | bim | 3.5 ± 0.1 |
| $[RuCym(en)(H_2O)]^{2+}$ | mim | < 2.6 |
| $[RuCym(en)(H_2O)]^{2+}$ | Py | < 1.5 |
| $[RuCym(phen)(H_2O)]^{2+}$ | bim | 5.6 ± 0.2 |
| $[RuCym(phen)(H_2O)]^{2+}$ | mim | 6.7 ± 0.2 |
| $[RuCym(phen)(H_2O)]^{2+}$ | Py | 4.3 ± 0.2 |
| $[RuCym(8HQH_{-1})(H_2O)]^{+}$ | bim | 2.9 ± 0.1 |
| $[RuCym(8HQH_{-1})(H_2O)]^{+}$ | mim | < 2.6 |
| $[RuCym(8HQH_{-1})(H_2O)]^{+}$ | Py | 1.6 ± 0.2 |

# EXPERIMENTAL DATA TABLES FOR SINGLE-CRYSTAL XRD

**Table S3.** Experimental parameters for the single crystal X-ray diffraction data collection and CCDC-code of the synthesized complexes.

| **CCDC-code** | **Sample** | **Machine** | **Source** | **Temp.** | **Detector Distance** | **Time/ Frame** | **Frames** | **Frame width** |
|---|---|---|---|---|---|---|---|---|
| | | | | [K] | [mm] | [s] | | [°] |
| 2209815 | **6′** | Stoe Stadivari | Mo | 100 | 40 | 10 | 1804 | 0.5 |
| 2209816 | **4′** | Bruker X8 | Mo | 100 | 36 | 6 | 1347 | 0.5 |
| 2209817 | **2′** | Bruker D8 | Mo | 100 | 34 | 6 | 1805 | 0.5 |
| 2209818 | **3′** | Bruker X8 | Mo | 100 | 40 | 8.1 | 5595 | 0.5 |
| 2209819 | **1′** | Bruker X8 | Mo | 100 | 35 | 7 | 833 | 0.6 |
| 2209820 | **5′** | Bruker D8 | Mo | 100 | 35 | 3 | 1390 | 0.6 |

**Table S4.** Crystal data and structure refinement for complexes **1′-3′**.

| | **1′** | **2′** | **3′** |
|---|---|---|---|
| Empirical formula | $C_{18}H_{29}F_6N_4O_6RhS_2$ | $C_{19}H_{28}F_6N_3O_6RhS_2$ | $C_{76}H_{73}B_2Cl_4N_4Rh$ |
| Formula weight | 678.48 | 675.47 | 1308.71 |
| Temperature (K) | 100.0 | 100.0 | 100.0 |
| Crystal system, Space group | triclinic | triclinic | triclinic |
| Space group | P-1 | P-1 | P-1 |
| Unit cell dimensions | | | |
| a (Å) | 7.7751(4) | 7.8309(4) | 12.9421(9) |
| b (Å) | 11.5350(7) | 10.8399(5) | 12.9452(9) |
| c (Å) | 15.4437(9) | 15.7969(7) | 20.0369(13) |
| $\alpha$ (°) | 100.4691(17) | 89.5353(16) | 76.964(3) |
| $\beta$ (°) | 91.3163(17) | 85.0745(16) | 85.322(3) |
| $\gamma$ (°) | 103.4897(17) | 79.0821(16) | 86.772(4) |
| Volume ($Å^3$) | 1321.35(13) | 1311.76(11) | 3255.8(4) |
| Z | 2 | 2 | 2 |
| $\rho_{calc}$ ($g/cm^3$) | 1.705 | 1.710 | 1.335 |
| $\mu$ ($mm^{-1}$) | 0.887 | 0.892 | 0.474 |
| F(000) | 688.0 | 684.0 | 1360.0 |
| Crystal size (mm) | 0.13×0.1× 0.07 | 0.423×0.227×0.08 | 0.3×0.11×0.08 |
| Radiation | Mo-$K_\alpha$ ($\lambda$=0.71073 Å) | | |
| 2Θ range for data collection (°) | 5.378-54.206 | 4.602-71.378 | 4.432-50.7 |
| Index ranges | -9≤h≤9, -14≤k≤14, -19≤l≤19 | -12≤h≤12, -17≤k≤17, -25≤l≤23 | -15≤h≤15, -15≤k≤15, -23≤l≤24 |
| Reflections collected | 14736 | 58577 | 97481 |
| Independent reflections | 5816 [$R_{int}$=0.0269, $R_{sigma}$=0.0308] | 12115 [$R_{int}$=0.0303, $R_{sigma}$=0.0271] | 11943 [$R_{int}$=0.0804, $R_{sigma}$=0.0482] |
| Data / restraints / parameters | 5816 /36 /364 | 12115 / 0 / 339 | 11943 / 0 / 790 |
| Goodness-of-fit on $F^2$ | 1.030 | 1.073 | 1.089 |
| Final *R* indices [$I$>2$\sigma$($I$)] | $R_1$=0.0331, $wR_2$=0.0788 | $R_1$=0.0265, $wR_2$=0.0571 | $R_1$=0.0452, $wR_2$=0.1179 |
| R indices (all data) | $R_1$=0.0361, $wR_2$=0.0809 | $R_1$=0.0328, $wR_2$=0.0592 | $R_1$=0.0501, $wR_2$=0.1220 |
| Largest diff. peak and hole (e×$Å^{-3}$) | 1.64 / -1.04 | 0.95 / -1.08 | 1.50 / -1.67 |

**Table S5.** Crystal data and structure refinement for complexes **4′-6′**.

| | **4′** | **5′** | **6′** |
|---|---|---|---|
| Empirical formula | $C_{30}H_{32}F_6N_3O_7RhS_2$ | $C_{48}H_{51}BN_3O_2Rh$ | $C_{48}H_{46}BN_2ORh$ |
| Formula weight | 827.61 | 815.64 | 780.59 |
| Temperature (K) | 100.0 | 100.0 | 100.0 |
| Crystal system, Space group | monoclinic | monoclinic | monoclinic |
| Space group | $P2_1/c$ | $P2_1/n$ | $P2_1/c$ |
| Unit cell dimensions | | | |
| a (Å) | 13.0591(11) | 11.8020(5) | 29.1880(10) |
| b (Å) | 13.5115(9) | 27.8595(9) | 11.8065(3) |
| c (Å) | 18.9926(15) | 12.2552(9) | 24.8781(10) |
| $\alpha$ (°) | 90 | 90 | 90 |
| β (°) | 102.252(3) | 90.8912(11) | 114.956(3) |
| γ (°) | 90 | 90 | 90 |
| Volume ($Å^3$) | 3274.9(4) | 4029.0(4) | 7772.7(5) |
| Z | 4 | 4 | 8 |
| $\rho_{calc}$ ($g/cm^3$) | 1.679 | 1.345 | 1.334 |
| μ ($mm^{-1}$) | 0.735 | 0.467 | 0.478 |
| F(000) | 1680.0 | 1704.0 | 3248.0 |
| Crystal size (mm) | 0.22×0.2×0.03 | 0.3×0.07×0.07 | 0.28×0.2×0.06 |
| Radiation | Mo-$K_\alpha$ ($\lambda$=0.71073 Å) | | |
| 2Θ range for data collection (°) | 3.728-50.69 | 4.524–60.092 | 3.274-51.364 |
| Index ranges | -14≤h≤15, -15≤k≤16, -22≤l≤22 | -16≤h≤16, -39≤k≤37, -17≤l≤17 | -35≤h≤35, -14≤k≤14, -30≤l≤25 |
| Reflections collected | 28186 | 144915 | 83725 |
| Independent reflections | 5887 [$R_{int}$=0.0506, $R_{sigma}$=0.0447] | 11780 [$R_{int}$=0.0628, $R_{sigma}$=0.0249] | 14760 [$R_{int}$=0.1697, $R_{sigma}$=0.1700] |
| Data / restraints / parameters | 5887 / 4 / 469 | 11780 / 0 / 504 | 14760 / 0 / 966 |
| Goodness-of-fit on $F^2$ | 1.046 | 1.074 | 0.953 |
| Final *R* indices [$I$>2σ($I$)] | $R_1$=0.0442, $wR_2$=0.1065 | $R_1$=0.0316, $wR_2$=0.0641 | $R_1$=0.0661, $wR_2$=0.1492 |
| R indices (all data) | $R_1$=0.0534, $wR_2$=0.1133 | $R_1$=0.0404, $wR_2$=0.0697 | $R_1$=0.1304, $wR_2$=0.1676 |
| Largest diff. peak and hole (e×$Å^{-3}$) | 0.90 / -0.80 | 0.67 / -0.75 | 2.26 / -1.31 |

# MIC VALUES FOR ALL COMPOUNDS, pH = 5–8

**Table S6.** Minimal inhibition concentration (MIC) (μM) of premixed complexes, bidentate and monodentate ligands on Gram-positive *S. aureus* bacterial strains after one day.

| MIC (μM) | *S. aureus*, sensitive | | | | *S. aureus*, resistant | | | |
|---|---|---|---|---|---|---|---|---|
| pH = | 5 | 6 | 7 | 8 | 5 | 6 | 7 | 8 |
| mim | Bacterial cell proliferation was inhibited at that pH. | | >100 | >100 | >100 | >100 | >100 | >100 |
| Py | | | >100 | >100 | >100 | >100 | >100 | >100 |
| econ | | | 0.78 | 0.19 | 25 | 12.5 | 6.25 | 3.125 |
| zol | | | >100 | >100 | >100 | >100 | >100 | >100 |
| phen | | | >100 | >100 | 50 | >100 | >100 | >100 |
| 8HQ | | | 25 | 25 | 100 | 6.25 | 6.25 | 6.25 |
| [RhCp*(phen)Cl]Cl | | | >100 | >100 | >100 | >100 | >100 | >100 |
| +mim | | | >100 | >100 | >100 | >100 | >100 | >100 |
| +Py | | | >100 | >100 | >100 | >100 | >100 | >100 |
| +econ | | | 0.78 | 1.56 | 25 | 12.5 | 3.125 | 3.125 |
| [RhCp*(8HQH$_{-1}$)Cl] | | | 12.5 | 12.5 | 50 | 12.5 | 12.5 | 12.5 |
| +mim | | | 12.5 | 12.5 | 50 | 25 | 12.5 | 12.5 |
| +econ | | | 0.39 | 0.39 | 12.5 | 6.25 | 3.125 | 3.125 |
| +zol | | | 12.5 | 25 | 50 | 50 | 12.5 | 12.5 |

**Table S7.** Minimal inhibition concentration (MIC) (μM) of premixed complexes, bidentate and monodentate ligands on Gram-negative *E. coli* bacterial strains after one day.

| MIC (μM) | *E. coli*, sensitive | | | | *E. coli*, resistant | | | |
|---|---|---|---|---|---|---|---|---|
| pH= | 5 | 6 | 7 | 8 | 5 | 6 | 7 | 8 |
| mim | >100 | >100 | >100 | >100 | >100 | >100 | >100 | >100 |
| Py | >100 | >100 | >100 | >100 | >100 | >100 | >100 | >100 |
| econ | >100 | >100 | >100 | >100 | >100 | >100 | >100 | >100 |
| zol | >100 | >100 | >100 | >100 | >100 | >100 | >100 | >100 |
| phen | 50 | 50 | 50 | 25 | 50 | 50 | 50 | 50 |
| 8HQ | >100 | >100 | >100 | >100 | >100 | >100 | >100 | >100 |
| [RhCp*(phen)Cl]Cl | >100 | >100 | >100 | >100 | >100 | >100 | >100 | >100 |
| +mim | >100 | >100 | >100 | >100 | >100 | >100 | >100 | >100 |
| +Py | >100 | >100 | >100 | >100 | >100 | >100 | >100 | >100 |
| +econ | >100 | >100 | >100 | >100 | >100 | >100 | >100 | >100 |
| [RhCp*(8HQH$_{-1}$)Cl] | >100 | >100 | >100 | >100 | >100 | >100 | >100 | >100 |
| +mim | >100 | >100 | >100 | >100 | >100 | >100 | >100 | >100 |
| +econ | 25 | 25 | 25 | 25 | 50 | 100 | 100 | 50 |
| +zol | >100 | >100 | >100 | >100 | >100 | >100 | >100 | >100 |

# REFERENCES


1. Wöckel, S.; Plessow, P.; Schelwies, M.; Brinks, M.K.; Rominger, F.; Hofmann, P.; Limbach, M. Alcohol Amination with Aminoacidato Cp*Ir(III)-Complexes as Catalysts: Dissociation of the Chelating Ligand during Initiation. *ACS Catal.* **2014**, *4*, 152–161. https://doi.org/10.1021/cs4009418
2. Babak, M.V.; Meier , S.M.; Legin, A.A.; Adib Razavi, M.S.; Roller, A.; Jakupec , M.A.; Keppler , B.K.; Hartinger, C.G. Am(m)ines Make the Difference: Organoruthenium Am(m)ine Complexes and Their Chemistry in Anticancer Drug Development. *Chem. Eur. J.* **2013**, *19*, 4308–4318. https://doi.org/10.1002/chem.201202657
3. Wang, F.; Habtemariam, A.; van der Geer, E.P.; Fernández, R.; Melchart, M.; Deeth, R.J.; Aird, R.; Guichard, S.; Fabbiani, F.P.; Lozano-Casal, P.; Oswald, I.D.; Jodrell, D.I.; Parsons, S.; Sadler, P.J. Controlling ligand substitution reactions of organometallic complexes: tuning cancer cell cytotoxicity. *Proc. Natl. Acad. Sci. U. S. A.* **2005**, *102*, 18269–18274. https://doi.org/10.1073/pnas.0505798102
4. Du, J.; Zhang, E.; Zhao, Y.; Zheng, W.; Zhang, Y.; Lin, Y.; Wang, Z.; Luo, Q.; Wu, K.; Wang, F. Discovery of a dual-targeting organometallic ruthenium complex with high activity inducing early stage apoptosis of cancer cells. *Metallomics* **2015**, *7* 1573–1583. https://doi.org/10.1039/C5MT00122F
5. Zhang, W.-Y.; Bridgewater, H.E.; Banerjee, S.; Soldevila-Barreda, J.J.; Clarkson, G.J.; Shi, H.; Imberti, C.; Sadler, P.J. Ligand-Controlled Reactivity and Cytotoxicity of Cyclometalated Rhodium(III) Complexes. *Eur. J. Inorg. Chem.* **2019**, *2020*, 1052–1060. https://doi.org/10.1002/ejic.201901055
6. Betanzos-Lara, S.; Salassa, L.; Habtemariam, A.; Novakova, O.; Pizarro, A.M.; Clarkson, G.J.; Liskova, B.; Brabec, V.; Sadler, P.J. Photoactivatable Organometallic Pyridyl Ruthenium(II) Arene Complexes. *Organometallics* **2012**, *31*, 3466–3479, https://doi.org/10.1021/om201177y
7. Battistin, F.; Balducci, G.; Wei, J.; Renfrew, A.K.; Alessio, E. Photolabile Ru Model Complexes with Chelating Diimine Ligands for Light-Triggered Drug Release. *Eur. J. Inorg. Chem.* **2018**, *2018*, 1469–1480, https://doi.org/10.1002/ejic.201701392
8. Liu, Z.; Romero-Canelón, I.; Habtemariam, A.; Clarkson, G.J.; Sadler, P.J. Potent Half-Sandwich Iridium(III) Anticancer Complexes Containing C∧N-Chelated and Pyridine Ligands. *Organometallics* 2014, *33*, 5324–5333. https://doi.org/10.1021/om500644f
9. Martinez, A.; Carreon, T.; Iniguez, E.; Anzellotti, A.; Sanchez, A.; Tyan, M.; Sattler, A.; Herrera, L.; Maldonado, R.A.; Sanchez-Delgado, R.A. Searching for New Chemotherapies for Tropical Diseases: Ruthenium–Clotrimazole Complexes Display High in Vitro Activity against Leishmania major and Trypanosoma cruzi and Low Toxicity toward Normal Mammalian Cells. *J. Med. Chem.* **2012**, *55*, 3867–3877. https://doi.org/10.1021/jm300070h
10. Liu, Z.; Romero-Canelón, I.; Qamar, B.; Hearn, J.M.; Habtemariam, A.; Barry, N.P.E.; Pizarro, A.M.; Clarkson, G.J.; Sadler, P.J. The Potent Oxidant Anticancer Activity of Organoiridium Catalysts. *Angew. Chem., Int. Ed.* **2014**, *53*, 3941–3946. https://doi.org/10.1002/anie.201311161
11. Hackl, C.M.; Legina, M.S.; Pichler, V.; Schmidlehner, M.; Roller, A.; Dömötör, O.; Enyedy, É.A.; Jakupec, M.A.; Kandioller, W.; Keppler, B.K. Thiomaltol-Based Organometallic Complexes with 1-Methylimidazole as Leaving Group: Synthesis, Stability, and Biological Behavior. *Chem. Eur. J.* **2016**, *22*, 17269–17281. https://doi.org/10.1002/chem.201603206
12. Schuecker, R.; John, R.O.; Jakupec, M.A.; Arion, V.B.; Keppler, B.K. Water-Soluble Mixed-Ligand Ruthenium(II) and Osmium(II) Arene Complexes with High Antiproliferative Activity. *Organometallics* **2008**, *27*, 6587–6595. https://doi.org/10.1021/om800774t
13. Enyedy, É.A.; Mészáros, J.P.; Dömötör, O.; Hackl, C.M.; Roller, A.; Keppler, B.K.; Kandioller, W. Comparative solution equilibrium studies on pentamethylcyclopentadienyl rhodium complexes of 2,2′-bipyridine and ethylenediamine and their interaction with human serum albumin. *J. Inorg. Biochem.* **2015**, *152*, 93–103. https://doi.org/10.1016/j.jinorgbio.2015.08.025
14. Dömötör, O.; Pape, V.F.S.; May, N.V.; Szakács, G.; Enyedy, É.A. Comparative solution equilibrium studies of antitumor ruthenium($\eta^6$-*p*-cymene) and rhodium ($\eta^5$-$C_5Me_5$) complexes of 8-hydroxyquinolines. *Dalton Trans.* **2017**, *46*, 4382–4392. https://doi.org/10.1039/C7DT00439G
15. Mészáros, J.P.; Pape, V.F.S.; Szakács, G.; Németi, G.; Dénes, M.; Holczbauer, T.; May, N.V.; Enyedy, É.A. Half-sandwich organometallic Ru and Rh complexes of (N,N) donor compounds: Effect of ligand methylation on solution speciation and anticancer activity. *Dalton Trans.* **2021**, *50*, 8218–8231. https://doi.org/10.1039/D1DT00808K
